# HprK_Xcc_ is a serine kinase that regulates virulence in the Gram‐negative phytopathogen *Xanthomonas campestris*

**DOI:** 10.1111/1462-2920.14740

**Published:** 2019-07-30

**Authors:** Rui‐Fang Li, Ping Cui, Ping‐Zhen Wei, Xing‐Yan Liu, Ji‐Liang Tang, Guang‐Tao Lu

**Affiliations:** ^1^ State Key Laboratory for Conservation and Utilization of Subtropical Agro‐bioresources College of Life Science and Technology, Guangxi University Nanning Guangxi China; ^2^ Guangxi Key Laboratory of Biology for Crop Diseases and Insect Pests Plant Protection Research Institute, Guangxi Academy of Agricultural Sciences Nanning Guangxi China

## Abstract

The HprK serine kinase is a component of the phosphoenolpyruvate phosphotransferase system (PTS) of bacteria that generally regulates catabolite repression through phosphorylation/dephosphorylation of the PTS protein PtsH at a conserved serine residue. However, many bacteria do not encode a complete PTS or even have an HprK homologue. *Xanthomonas campestris* pv. *campestris* (*Xcc*) is a pathogen that cause black rot disease in crucifer plants and one of the few Gram‐negative bacteria that encodes a homologue of HprK protein (herein HprK_Xcc_). To gain insight into the role of HprK_Xcc_ and other PTS‐related components in *Xcc* we individually mutated and phenotypically assessed the resulting strains. Deletion of *hprK*_*Xcc*_ demonstrated its requirement for virulence and other diverse cellular processes associated including extracellular enzyme activity, extracellular‐polysaccharide production and cell motility. Global transcriptome analyses revealed the HprK_Xcc_ had a broad regulatory role in *Xcc*. Additionally, through overexpression, double gene deletion and transcriptome analysis we demonstrated that *hprK*_*Xcc*_ shares an epistatic relationship with *ptsH*. Furthermore, we demonstrate that HprK_Xcc_ is a functional serine kinase, which has the ability to phosphorylate PtsH. Taken together, the data illustrates the previously unappreciated global regulatory role of HprK_Xcc_ and previously uncharacterized PTS components that control virulence in this pathogen.

## Introduction

*Xanthomonas campestris* pv. *campestris* (*Xcc*) is an aerobic, Gram‐negative rod bacterium that is known to infect plants. This phytopathogen causes black rot disease in almost all the members of the crucifer family (*Brassicaceae*) which includes vegetables such as broccoli, Brussel sprouts, cabbage, cauliflower, kale, mustard, radish and oil seed rape (Vicente and Holub, 2013). *Xcc* infects host plants *via* wounds or hydathodes. After infection, the bacterial cells multiply in the intercellular spaces, spreading via vascular system, and leading to the development of disease symptoms (Chan and Goodwin, 1999). The virulence of *Xcc* toward plants depends on a number of factors, including adhesion, motility, biofilm formation, secretion of cell wall‐degrading enzymes, extracellular‐ and lipo‐polysaccharides and type III effector protein secretion (Büttner and Bonas, 2010; Ryan *et al*., 2011). It is known that virulence factors in *Xcc* are tightly regulated by many different systems but the two which have gained most notoriety are the diffusible signal factor (DSF) signal‐dependent quorum‐sensing system (Tang *et al*., 1991), and the hypersensitive reaction and pathogenicity (*hrp*) regulatory system for type III secretion (Lindgren *et al*., 1986). Despite detailed studies of virulence regulation in *Xcc*, there are many regulatory pathways that contribute to virulence and disease that have yet to be characterized.

The phosphoenolpyruvate phosphotransferase system (PTS) is a multicomponent phosphotransfer cascade that has been shown in many bacteria to mediate transport and phosphorylation of selected sugars, such as glucose, sucrose, mannose and *N*‐acetylglucosamine (Deutscher *et al*., 2014). Phosphate enters the PTS through transfer from phosphoenolpyruvate to the first PTS component, the phosphoenolpyruvate‐protein phosphotransferase (enzyme I) encoded by *ptsI* gene. This phosphoenolpyruvate‐protein phosphotransferase in turn transfers the phosphate group to another component of the PTS, histidine‐containing phosphocarrier protein (HPr) encoded by *ptsH* gene. Many bacterial genomes encode a protein homologous to PtsH termed FPr, which is preferred for transport of fructose through the PTS. PtsH and FPr transfer phosphate to a number of enzymes, which are multi‐subunit, membrane‐associated complexes that carry out transport and phosphorylation of specific PTS substrates. Given that transport of PTS substrates rapidly depletes the PTS of phosphorylated intermediates, the phosphorylation states of PTS components serve as cytoplasmic reporters of environmental nutrient availability.

Although the PTS system primary role has been shown to be in carbohydrate transport, PTS proteins in some bacteria carry out other regulatory functions in metabolism, potassium transport, chemotaxis biofilm formation and virulence (Deutscher *et al*., 2014; Saier, 2015). Depending on their phosphorylation state, which varies according to the availability of PTS substrates and the metabolic state of the cell, the four proteins (including PtsH) forming the PTS phosphorylation cascade can phosphorylate or interact with other non‐PTS proteins and regulate their activity (Deutscher *et al*., 2014).

Although the PtsH (or HPr) protein can be phosphorylated at residue His‐15 by phosphoenolpyruvate‐protein phosphotransferase, it can also be phosphorylated at its residue Ser‐46 by the HPr (Ser) kinase (HprK), which also has been shown to possess phosphatase activity in select bacteria such as *Enterococcus faecalis* and *Lactobacillus casei*, forming serine‐phosphorylated PtsH (P‐Ser‐PtsH) (Deutscher and Saier, 1983; Dossonnet *et al*., 2000; Poncet *et al*., 2004). Additional studies have shown that P‐Ser‐PtsH (or P‐Ser‐HPr) regulates carbohydrate metabolism via forming a complex with CcpA (catabolite control protein A). The CcpA/P‐Ser‐PtsH complex binds to specific operator sites *cre* (catabolite responsive element), preventing transcription of numerous catabolite‐regulated genes (Deutscher *et al*., 1995; Deutscher *et al*., 2001). P‐Ser‐PtsH has also been shown to play a role in inducer exclusion of Gram‐positive bacteria, in which it binds to components of carbohydrate‐specific ABC transporters and inhibits their activity (Dossonnet *et al*., 2000; Monedero *et al*., 2001). Additionally, P‐Ser‐PtsH also contributes to virulence in certain Gram‐positive pathogenic bacteria, e.g. *Clostridium difficile*, *Listeria monocytogenes* and *Streptococcus pneumonia* (Herro *et al*., 2005; Iyer *et al*., 2005; Antunes *et al*., 2011). Although much work has been carried out in the study of the function of HprK in Gram‐positive bacteria its role in Gram‐negative strains has been less well studied. Over the past decade, genome sequence analysis has revealed that many Gram‐negative bacteria also possess homologues of HprK, but appear to lack CcpA, providing no real insight into the role that HprK contributes in these organisms (Reizer *et al*., 1998; Hu and Saier, 2002; Stonestrom *et al*., 2005).

In *Xcc*, a functional frucose‐specific PTS system has been identified and characterized (de Crécy‐Lagard *et al*., 1991). Despite these observations little further study has been carried out to understand the role of this system in *Xcc*. In the present study, we detail the assessment of *Xcc* 8004 sequenced genome which revealed that this bacterium possesses a cluster of genes encoding homologues of the PTS proteins, including Enzyme I (PtsI, XC_1304), HPr (PtsH, XC_1305) and two EIIA‐like proteins EIIA^Man^ (PtsN^Man^, XC_1306) and EIIA^Ntr^ (PtsN^Ntr^, XC_1309). Interestingly, the cluster also encodes an PtsH (HPr) (Ser) kinase homologue (XC_1308, herein named HprK_Xcc_), but no the CcpA homologue. To gain insight into the role of these components in *Xcc*, the five PTS‐related genes were individually deleted and phenotypically assessed. The analysis revealed that HprK_Xcc_ is required for virulence and other diverse cellular processes associated with virulence, including extracellular enzyme activity, extracellular‐polysaccharide production, cell motility and tolerance to various stresses. Focusing on HprK_Xcc_, transcription analysis revealed that this protein is a global regulator that controls at least 256 genes under the conditions tested. Moreover, our data also showed that PtsH protein is required for the regulatory function of HprK_Xcc_. These results illustrate the complexity of regulation in *Xcc* by previously uncharacterized PTS components and underscore the importance of HprK_Xcc_ in the control of virulence functions. Furthermore, to our knowledge, this is the first description of an HprK (Ser) kinase protein playing a global regulatory role in virulence related functions.

## Results

### *The* Xcc *genome encodes a partial PTS system where HprK_Xcc_ is required for full virulence*

**Figure 1:**
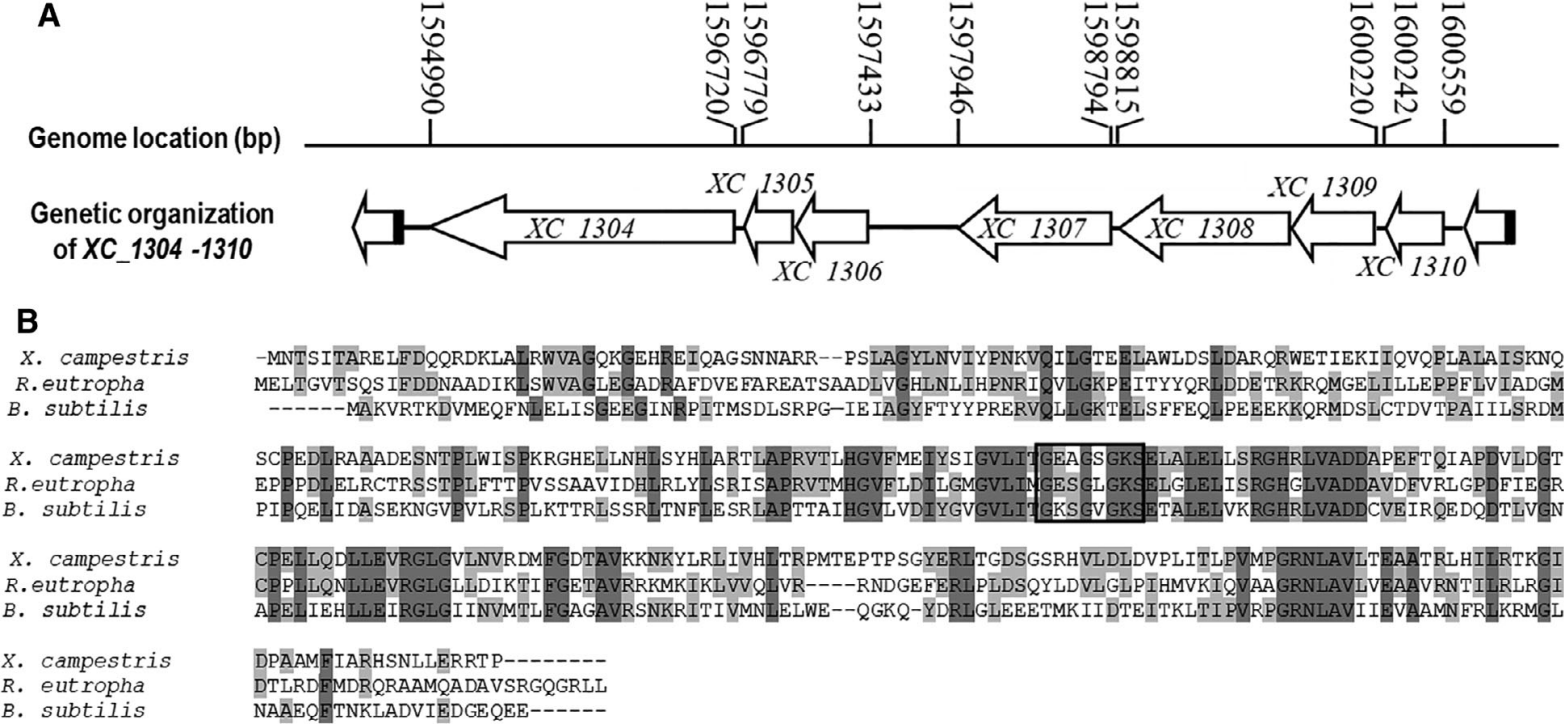
*Xcc* genome encodes elements of a PTS and a HprK homologue. A. Genetic and physical map of *XC1304*‐*1310* in the genome of *Xcc*. The positions and orientations of the gene *XC1304*‐*1310* are shown; arrows indicate length, location and orientation of the genes, lines indicate the intergenic sequences. *XC_1304* (*ptsI*) encodes a phosphotransferase system enzyme I; *XC_1305* (*ptsH*) encodes a histidine‐containing phosphocarrier protein (HPr); *XC_1306* encodes a hypothetical protein which contains a fructose IIA component domain and P‐loop ATPase protein family domain; *XC_1307* encodes a conserved hypothetical protein recently characterized as an ATPase/phosphatase bi‐functional enzyme (Cui *et al*., 2018); *XC_1308* (*hprK*_*Xcc*_) encodes a HPr kinase; *XC_1309* (*ptsN*) encodes a nitrogen regulatory IIA protein, and *XC_1310* (*rpoN*) encodes a sigma‐54 modulation protein. B. Amino acid sequence pairwise alignments using Vector NTI showed that the protein encoded by *XC_1308* shares identity to the characterized HPr (Ser) kinase/phosphorylase (HprK) in *Ralstonia eutropha* (accession number Q0KEN8) and *Bacillus subtilis* (accession number O34483). Conserved residues are shown with grey and light grey background. The putative Walker A motifs are indicated by a square box. Walker A is generally a consensus sequence of (A/G)X4GK(T/S), which is centered at a loop between a β‐strand and an α‐helix.

As a first step to characterizing the PTS system in *Xcc*, we examined the genome of strain 8004 (accession number CP000050). This revealed that the bacterium harbours an incomplete PTS gene cluster (*XC_1304–1309*) that encoded PtsI (Enzyme I), PtsH (HPr), PtsN^Man^/PtsN^Ntr^ (two IIA‐like proteins), and HprK (HPr [Ser] kinase) (Fig. 1). The cluster did not encode CcpA, EIIB and EIIC homologues which are seen in other bacterial strains. To evaluate the function of these putative PTS‐related genes in *Xcc*, deletion mutants of *ptsI* (*XC_1304*), *ptsH* (*XC_1305*), *ptsN* ^*Man*^ (*XC1306*), *ptsN* ^*Ntr*^ (*XC_1309*) and *hprK*_*Xcc*_ (*XC_1308*) were constructed by using the suicide vector pK18mob*sacB* (see Methods) and the respective strains were designated ΔptsI, ΔptsH, ΔptsN^Man^, ΔptsN^Ntr^ and ΔhprK_Xcc_ (Supporting Information Table S1). Simultaneously, complemented strains were constructed by introducing the recombinant plasmid pLAFR6, which carried the gene of interest along with its promoter, into the target strain (see Methods).

To examine if the presence of homologues of *ptsI*, *ptsH*, *ptsN* ^*Man*^, *ptsN* ^*Ntr*^ and *hprK*_*Xcc*_ contribute to sugar uptake in *Xcc* as seen in other bacterial strains we assessed each mutant's ability to grow on non‐carbohydrate minimal medium (NCM) agar plates supplemented with a variety of sugars as sole carbon source. Results revealed that the colonies of strains ΔptsI, ΔptsH, ΔptsN^Man^ and ΔptsN^Ntr^ were similar to that of the wild‐type, indicating that mutation in these genes had no obvious impact on sugar uptake or transport under the conditions tested. However, the ΔhprK_Xcc_ strain when grown produced smaller colonies when compared to the wild‐type strain (Supporting Information Fig. S1A). This prompted us to examine the growth characteristics of this strain in rich nutritional medium NYG and minimal medium MMX (Supporting Information Fig. S1B,C). The ΔhprK_Xcc_ mutant demonstrated initial slower growth at early exponential phase compared to that of the wild‐type strain. Interestingly, the doubling times of the ΔhprK_Xcc_ mutant and wild‐type strain were similar during the exponential period, they were ~2.2 h in NYG and 4.4 h in MMX respectively, indicating that a mutation in HprK_Xcc_ does not affect the growth of *Xcc* in standard media on exponential phase.

**Figure 2:**
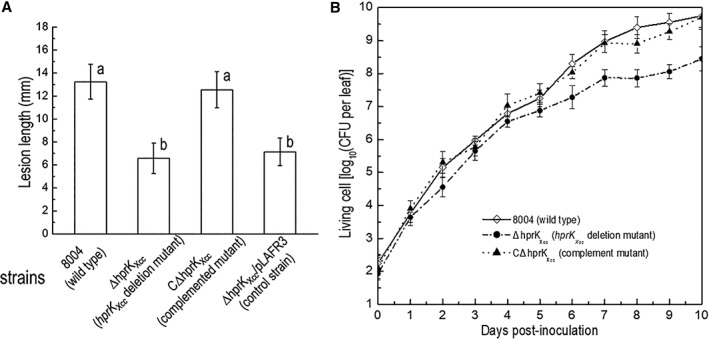
HprK_Xcc_ is important for full virulence in *Xcc*. A. Mean lesion lengths caused by different *Xcc* strains. *Xcc* strains cells were resuspended in 10 mM sodium phosphate buffer at the concentration of 1 × 10^7^ CFU (colony forming units) ml^−1^ (OD_600_ of 0.01). Chinese radish (*Raphanus sativus*) leaves were cut with scissors dipped in the bacterial suspensions. Lesion lengths were scored at 10 days post‐inoculation. Values given are the means and standard deviations from 15 measurements. The different letters on each column indicate significant differences at *P* = 0.05 by *t‐*test. B. Bacterial populations of *Xcc* strains in host plant leaf tissue. Inoculated leaves for each strain were taken daily and homogenized in sterile water. The homogenates were diluted and plated on NYG plates. Bacterial CFUs were counted after incubation for 3 days. Data are the means and standard deviations from three replicates.

In order to explore the impact that these mutations had on virulence of *Xcc*, these strains were tested in the host plant Chinese radish using a leaf clipping assay (see Methods). As shown in Fig. 2, the ΔhprK_Xcc_ strain produced a mean lesion length in radish of 6.58 mm which was significantly less disease (*P* = 0.05 by *t‐*test) compared to wild‐type (Fig. 2A). However, the other mutants caused similar disease symptoms to the wild‐type (data not shown). Furthermore, the complemented strain CΔhprK_Xcc_ showed a mean lesion length of 12.5 mm, which was not significantly different from the lesions caused by the wild‐type strain (*P* = 0.05 by *t*‐test). Additionally, the empty vector pLAFR3 was also introduced into the ΔhprK_Xcc_ mutant, the resulting strain ΔhprK_Xcc_/pLAFR3 caused similar lesion length with the ΔhprK_Xcc_ mutant (Fig. 2A). These data indicate that *hprK*_*Xcc*_ is important for the virulence of *Xcc*. The growth of *Xcc* strains in the host plant was further estimated (see Methods). For the ΔhprK_Xcc_ mutant, the number of bacterial cells recovered from the infected leaves was similar to that of the wild‐type strain within 5 days post‐inoculation (Fig. 2B), indicating the *hprK*_*Xcc*_ does not influence growth of *Xcc in planta* in the invasive stage. However, the mutant grows slow compared to the wild‐type after 6 days post‐inoculation. At 10 days post‐inoculation, when the lesion length was measured, the mutant population decreased ~20‐fold compared to that of the wild‐type, indicating that mutation in HprK_Xcc_ reduces the *in planta* fitness during the symptom development.

### *HprK_Xcc_ regulates genes involved in virulence and various adaptation processes in* Xcc

To get a better understanding of the scope and regulatory role of HprK_Xcc_ in *Xcc* a set of global gene expression profiles were generated using transcriptome profiling. Here we explored the expression profile of the ΔhprK_Xcc_ mutant and wild‐type strain 8004 by using RNA‐seq analysis. For this experiment, *Xcc* strains were grown to the mid‐exponential phase (OD_600_ = 0.6) in medium NYG, which has been widely used in the studies of the morphology, biology and preservation of *Xcc* (see Methods). Following bacterial RNA extraction, library construction and sequencing differential gene expression analysis was conducted on the generated data (see Methods).

**Figure 3:**
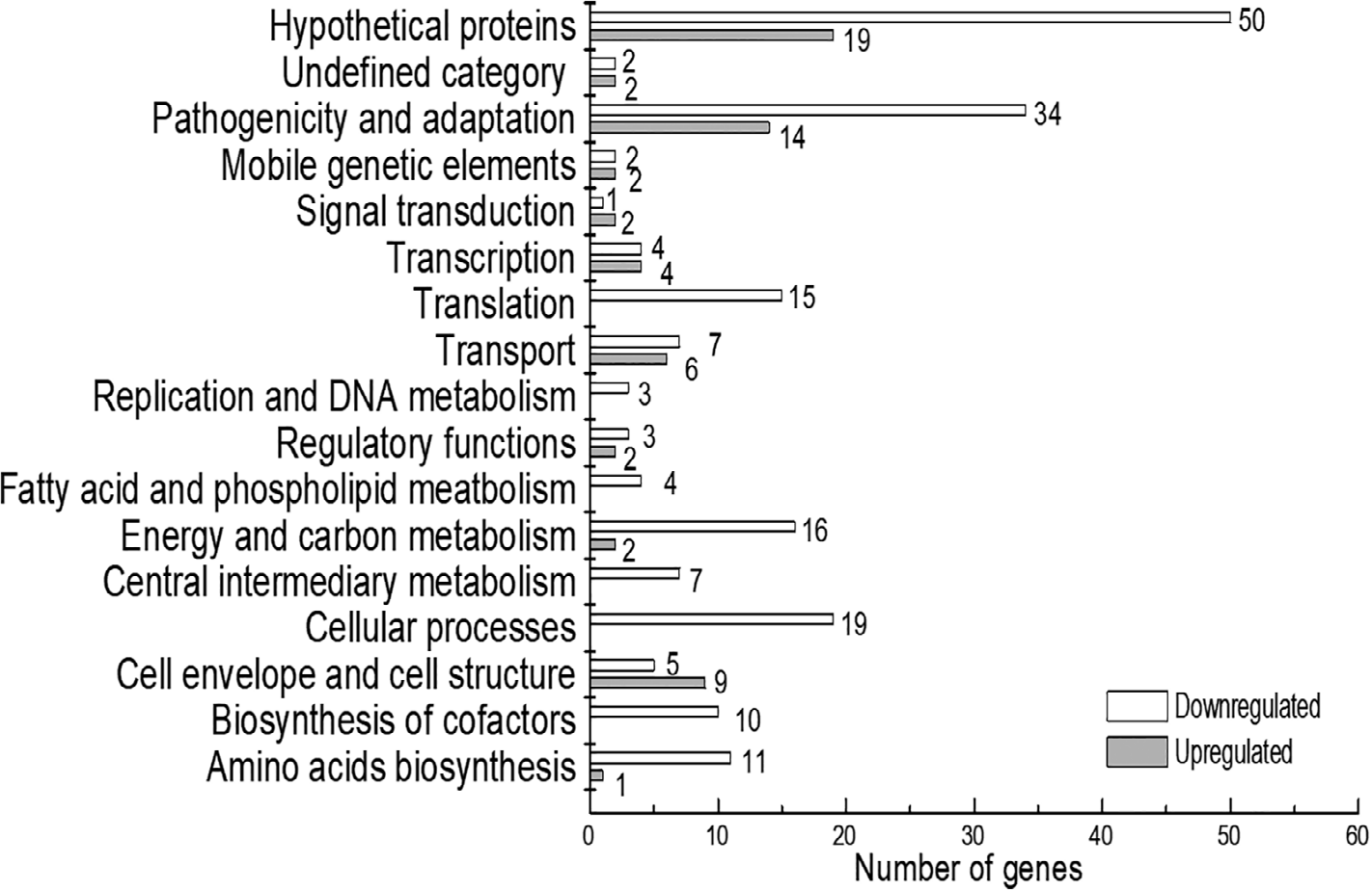
HprK_Xcc_ has a broad regulatory role in *Xcc*. Functional categories of differential expressed genes (DEGs) in *hprK*_*Xcc*_ mutant ΔhprK_Xcc_. Genome‐scale transcriptome profiling of *Xcc* strains cultured in nutrition rich medium NYG were investigated by RNA‐sequencing, and 256 genes were found differentially expressed by two‐fold or more in *hprK*_*Xcc*_ mutant (Table S2). These genes were broadly categorized according to their biological function (He *et al*., 2007).

Analysis revealed that a total of 256 genes, of the 4273 annotated protein‐coding genes in the genome of *Xcc* 8004 strain, were found to be influenced by HprK_Xcc_ under the conditions tested (Qian *et al*., 2005). Among genes that were altered in the HprK_Xcc_ mutant, 63 genes were upregulated (≥2‐fold) and 193 were downregulated (≤2‐fold) (Supporting Information Table S2). Functional clustering analysis, according to the annotation of *Xcc* 8004 genome, was carried out. One hundred eighty‐three genes were assigned to 15 functional categories but the remaining 73 genes encoded hypothetical proteins or have not been given a functional category to date (Fig. 3; Supporting Information Table S2) (Qian *et al*., 2005; He *et al*., 2007; Febrer *et al*., 2011). A total of 48 genes were identified to belong to the group of ‘pathogenicity and adaptation’, 19 to ‘cellular processes’ and 15 to ‘translation’ (Fig. 3; Supporting Information Table S2).

Notably, HprK_Xcc_ had a significant negative impact on genes that contribute to extracellular‐polysaccharide (EPS), extracellular enzymes, motility, stress tolerance (Fig. 3; Supporting Information Table S2). For example, both *XC_1658* and *XC_1659* or genes that encode the proteins involved in EPS synthesis, *XC_0738*, *XC_0745* and *XC_0748* encoded proteins involved in type II secretion system, *XC_3376* and *XC_3377* encode characterized extracellular proteases (Dow *et al*., 1993), *XC_0028* and *XC_0625*, *XC_1298*, *XC_3591* encode characterized cellulase and pectate lyases (Dow *et al*., 1989). Additionally, genes involved in pili‐dependent motility, chemotaxis and protein transporter were also influenced (Fig. 3; Supporting Information Table S2). To verify and validate the transcriptome data, several differentially expressed genes (DEGs) were selected and confirmed by using Semi‐quantitative reverse‐transcription polymerase chain reaction (RT‐PCR) (see Methods). These RT‐PCR tested genes represented those with a range of fold change of expression and of diverse functional classes. Results shown that expression of these selected genes was consistent with the data from the transcriptome analyses (Supporting Information Table S3).

### *HprK_Xcc_ is necessary in the regulation of extracellular enzyme activity, EPS production, cell motility and tolerance to environmental stress*

The above data revealed that HprK_Xcc_ appears to influence the expression of genes involved in wide‐ranging functions associated with virulence and pathogenesis in *Xcc*. To assess if HprK_Xcc_ has an impact on these functions at phenotypic level, we conducted series of phenotypic tests including extracellular enzyme production (including protease, endoglucanase, amylase and pectate lyase), EPS production, motility and the adaption to stress and antimicrobials.

**Figure 4:**
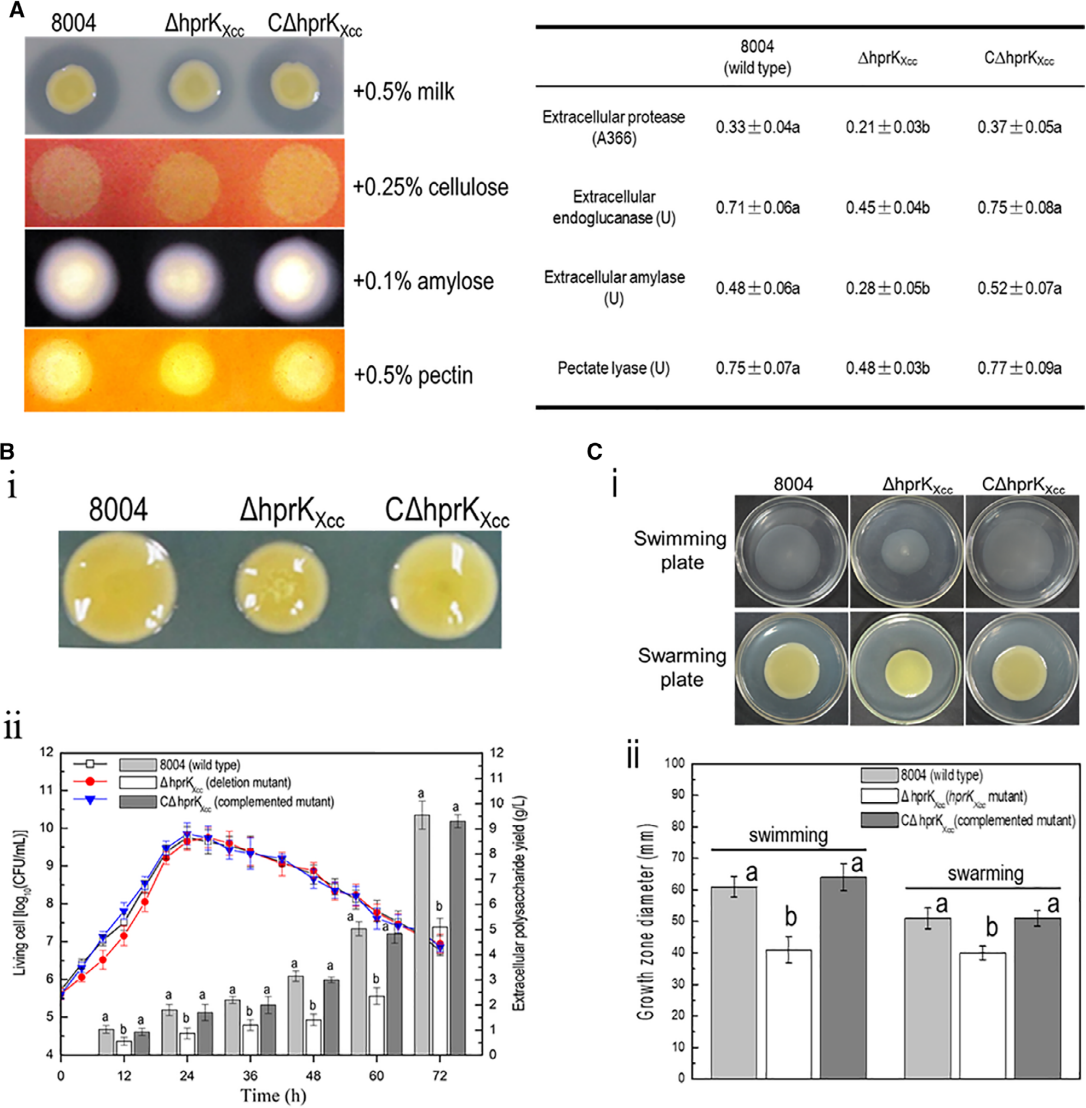
HrpK_Xcc_ is required for the regulation of phenotypes associated with virulence in *Xcc*. A. The level of extracellular enzymes produced by the ΔhprK_Xcc_ strain was significantly reduced compared with that of the wild‐type. An overnight culture (2 μl, OD_600_ = 1.0) of each *Xcc* strain was spotted onto a NYG plates containing 0.5% (wt/vol) skim milk (for protease), 0.25% (wt/vol) carboxymethylcellulose (for endoglucanase), 0.1% (wt/vol) starch (for amylase) or 0.5% pectin (for pectate lyase) and incubated at 28°C for 24 h (endoglucanase and amylase) or 48 h (protease and pectate lyase). Plates were stained when necessary. Zones of clearance around the spot due to the degradation of the substrate were photographed (left). To estimate quantitatively the activity of endoglucanase (cellulase), amylase, pectate lyase and protease, *Xcc* strains were cultured in NYG medium for 12 h and adjusted to the same concentration, and the level of activity was assessed and recorded (right). Data are the mean ± standard deviation of triplicate measurements; the different letters in each data column indicate significant differences at *P* = 0.05 by *t*‐test. The experiment was repeated twice and similar results were obtained. B. The *hprK*_*Xcc*_ mutant produces less EPS compared to the wild‐type. (i) Strains grown on NY agar plates supplemented with 2% glucose for 5 days. The representative colony morphology of *Xcc* strains was photographed. *hprK*_*Xcc*_ mutant displayed smaller colonies than the wild‐type strain, while the complemented strain CΔ*hprK*_*Xcc*_ formed normal wild‐type colonies (i). (ii) Time‐course of EPS production. *Xcc* strains were cultured in NY medium supplemented with 2% (wt/vol) glucose. EPS yield and bacterial growth were determined at 12 and 4 h intervals respectively, until 72 h. Bars represent the EPS yield. Curves represent bacterial growth, measured by counting bacterial CFU. Data are the means ± standard deviation of three replicates. The different letters on each column indicate significant differences at *P* = 0.05 by *t*‐test. C. The swimming and swarming motility of ΔhprK_Xcc_ strain was significantly reduced compared with that of the wild‐type. *Xcc* strains were stabbed into ‘swim’ (0.28% agar) medium followed by incubation at 28°C for 4 days and inoculated onto ‘swarm’ (0.6% agar) medium followed by a 3‐day incubation at 28°C. The representative colony morphology of *Xcc* strains were photographed (i), and colony diameters of each strain on the different media were measured (ii). Values given are the means ± standard deviations of triplicate measurements from a representative experiment, similar results were obtained in two other independent experiments. The different letters on each column indicate significant differences at *P* = 0.05 by *t*‐test. [Color figure can be viewed at http://wileyonlinelibrary.com]

To test the effect of HprK_Xcc_ on extracellular enzymes, ΔhprK_Xcc_ and wild‐type strain were compared when grown on the NYG agar plates containing skimmed milk (for protease), carboxymethylcellulose (for endoglucanase/cellulase), starch (for amylase), or pectin respectively (see Methods). Results revealed that the diameter of the zone of the ΔhprK_Xcc_ mutant on NYG plates containing skimmed milk, carboxymethylcellulose, or starch was smaller than the wild‐type strain (Fig. 4A). In tandem, the activities of these extracellular enzymes produced by the ΔhprK_Xcc_ and wild‐type strain were quantitatively estimated (see Methods). As shown in Fig. 4A, the activities of all tested enzymes produced by the ΔhprK_Xcc_ were significantly diminished compared to the wild‐type strain (*P* = 0.05 by *t*‐test). Moreover, activities of extracellular enzymes of the complemented mutant strain CΔhprK_Xcc_ showed no significant difference from that of the wild‐type.

To examine the EPS production, the ΔhprK_Xcc_ and wild‐type strains were grown on NY agar plates supplemented with 2% glucose for 5 days (see Methods). The ΔhprK_Xcc_ mutant displayed smaller colonies than the wild‐type (Fig. 4B‐i), suggesting that *hprK*_*Xcc*_ mutant might produce less EPS than the wild‐type. To quantitatively measure the EPS yield, strains were grown in NY liquid medium supplemented with 2% of glucose for 3 days, and EPS was extracted from the cultures (see Methods). As showed in Fig. 4B‐ii, the *hprK*_*Xcc*_ mutant produced ~49.5% less EPS than the wild‐type. In addition, the EPS yield of the complemented mutant strain showed no significant difference from that of the wild‐type.

The ability of the ΔhprK_Xcc_ strain to swim and swarm was also examined. To test swimming motility, *Xcc* strains were inoculated into swimming plates (0.28% agar) and incubated for 4 days. As shown in Fig. 4C, the mutant displayed severely weakened swimming ability compared to the wild‐type. As analysed by the *t*‐test, the mean radius of the ΔhprK_Xcc_ mutant was significantly shorter than that of the wild‐type (*P* = 0.05 by *t*‐test). Additionally, the swarming ability of the *hprK*_*Xcc*_ mutant was also examined, for this *Xcc* strains were inoculated into 0.6% agar plates and incubated for 3 days. Again, the *hprK*_*Xcc*_ mutant was significantly less motility than the wild‐type strain (Fig. 4C). Importantly, the complemented strain and the wild‐type strain were not significantly different in swimming and swarming plates. These combined data indicated that mutation in HprK_Xcc_ reduces the cell motilities.

To investigate if HprK_Xcc_ contributes to environmental stress adaptation, we determined the survival of the mutant ΔhprK_Xcc_, wild‐type strain 8004 and the complemented mutant strain CΔhprK_Xcc_ under various environmental conditions, including osmotic challenge (NaCl), sodium dodecyl sulfate (SDS), heavy metal stress (CdCl_2_) and the organic solvent phenol (see Methods). Results revealed that MICs of NaCl, SDS, phenol and heavy metal Cd^2+^ for *hprK*_*Xcc*_ mutant were obviously lower than those for the wild type (*P* = 0.05 by *t*‐test), while the MICs for wild‐type and complemented strain were almost identical (Supporting Information Fig. S2), indicating that tolerance of the *hprK*_*Xcc*_ mutant to these environmental stresses is reduced.

### *The gene* hprK_Xcc_ *shares an epistatic relationship with* ptsH

The above data revealed that PtsI, PtsH, PtsN^Man^ or PtsN^Ntr^ did not influence virulence or sugar uptake. However, the influence of these proteins may be masked by HprK_Xcc._ To explore the epistatic relation between the *hprK*_*Xcc*_ and *ptsI*, *ptsH*, *ptsN* ^*Man*^ or *ptsN* ^*Ntr*^ we generated a series of strains carrying double‐deletions (see Methods). These strains were designated ΔhprK_Xcc_ΔptsI, ΔhprK_Xcc_ΔptsH, ΔhprK_Xcc_ΔptsN^Man^ and ΔhprK_Xcc_ΔptsN^Ntr^. These strains were examined using the same phenotypic assays used to assess the ΔhprK_Xc*c*_ strain previously (virulence, extracellular enzymes production, EPS production and motility).

**Figure 5:**
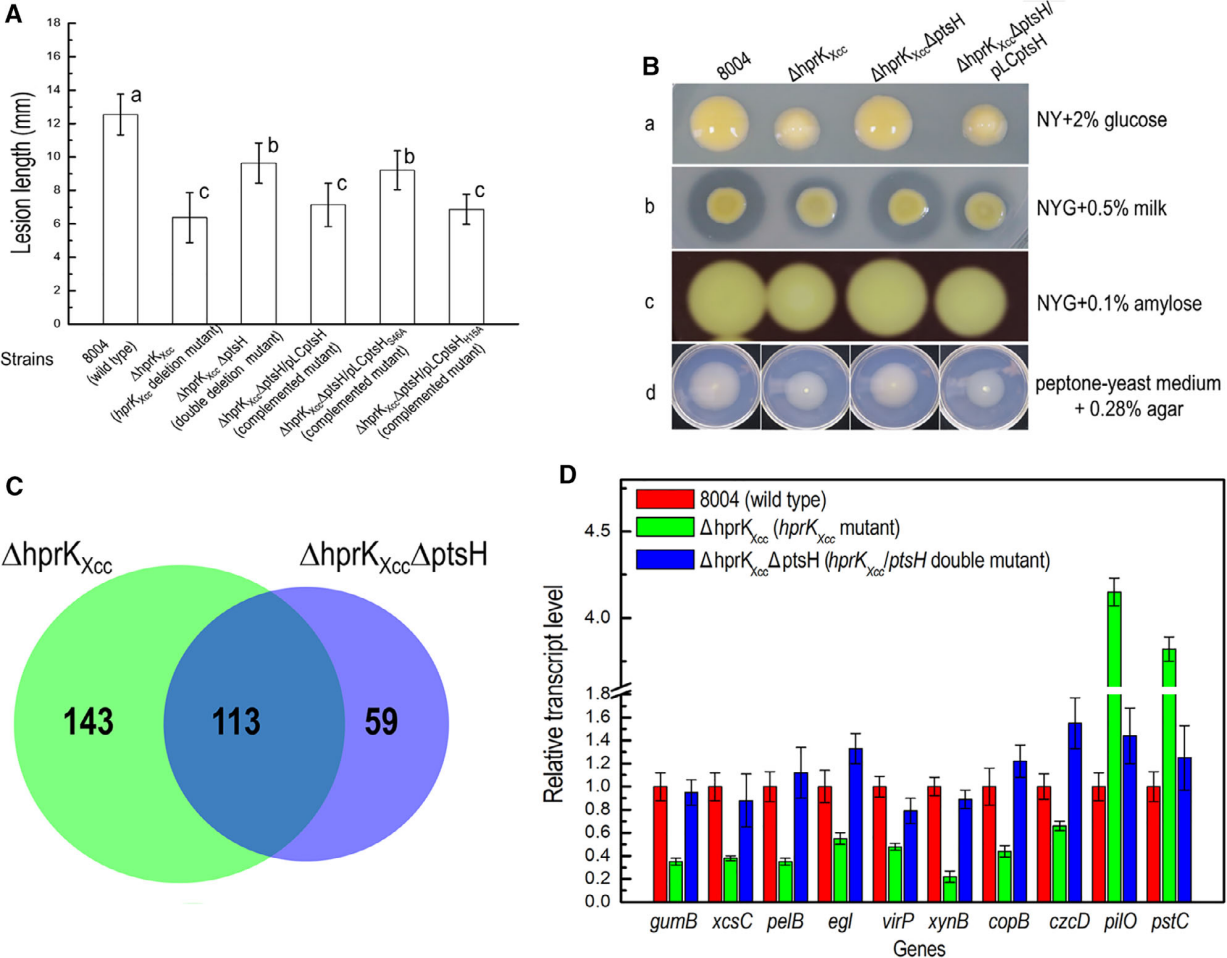
*hprK*_*Xcc*_ shares an epistatic relationship with *ptsH*. A. Mean lesion lengths caused by different *Xcc* strains. Lesion lengths were scored at 10 days post‐inoculation. Values given are the means and standard deviations from 15 measurements. The different letters on each column indicate significant differences at *P* = 0.05 by *t*‐test. B. The level of EPS production (a), activity of extracellular enzymes [protease (b) and amylase (c)] and cell motility (swimming) in different *Xcc* strains. C. Comparison of gene expression changes in the *hprK*_*Xcc*_ and *hprK*_*Xcc*_/*ptsH* deletion mutant. Venn diagrams showing the overlap of genes whose expression is upregulated and downregulated in *hprK*_*Xcc*_ and *hprK*_*Xcc*_/*ptsH* deletion mutant backgrounds. D. qRT‐PCR verification of differently expressed genes in the *hprK*_*Xcc*_ and *hprK*_*Xcc*_/*ptsH* deletion mutant backgrounds compared to wild‐type. The expression of these selected genes was influenced in the *hprK*_*Xcc*_ mutant but not in *hrpK*_*Xcc*_/*ptsH* double mutant. [Color figure can be viewed at http://wileyonlinelibrary.com]

Phenotypic tests showed that the *hprK*_*Xcc*_/*ptsI*, *hprK*_*Xcc*_/*ptsN* ^*Man*^ or *hprK*_*Xcc*_/*ptsN* ^*Nt*r^ double‐deletion mutant displayed phenotypes similar to that of the *hprK*_*Xcc*_ mutant (data not shown). However, the ΔhprK_Xcc_ΔptsH double mutant displayed similar wild‐type phenotypes (Fig. 5A,B). In order to verify this result, a complemented strain for ΔhprK_Xcc_ΔptsH was constructed. This was achieved by introducing the plasmid pLCptsH, which derived from a 270‐bp DNA fragment of the *ptsH* ORF sequence cloned into the plasmid pLAFR3, into the mutant strain ΔhprK_Xcc_ΔptsH (see Methods). The complemented strain ΔhprK_Xcc_ΔptsH/pLCptsH revealed similar phenotypes to that of the ΔhprK_Xcc_ mutant (Fig. 5A,B). These combined data suggest that the *ptsH* gene has an epistatic relationship with the *hprK*_*Xcc*_ gene. This view was strengthened by examination of transcriptome of the double mutant ΔhprK_Xcc_ΔptsH grown to the mid‐exponential phase (OD_600_ = 0.6) in medium NYG and compared with the data generated for the ΔhrpK_Xcc_ mutant (see Methods). Analysis revealed that 172 genes are differentially expressed in *hrpK*_*Xcc*_/*ptsH* double mutant compared to the wild‐type. These DEGs were broadly categorized according to their biological function (Supporting Information Table S2, Fig. S3). To verify the transcriptome data, semi‐quantitative RT‐PCR was performed to analyse the relative expression levels of several selected genes. Expression of these selected genes was consistent with the data from the transcriptome analyses (Supporting Information Table S4). Among these DEGs, 113 genes are overlapped with that in *hprK*_*Xcc*_ mutant, implying the expression of 143 genes seen to be influenced in the ΔhrpK_Xcc_ mutant were restored toward wild‐type in the ΔhprK_Xcc_ΔptsH double mutant background (Fig. 5C). To confirm this, several genes (e.g. *gumB*, *xcsC*, *pelB*, *egl*) were selected and assessed by using quantitative real‐time PCR (qRT‐PCR). Results demonstrated that the expression of the selected gene was consistent with the data from the transcriptome analyses (Fig. 5D, Supporting Information Table S2). Among these 143 genes, lots contribute to the virulence factors e.g. EPS production, extracellular enzymes production/secrection, motility and stress tolerance. This appears consistent with the phenotypes we observed.

In Gram‐positive bacteria, many PtsH (also called HPr) proteins have been shown to be phosphorylated on two different residues. In *Bacillus subtilis* EI phosphorylates PtsH at the histidine‐15 residue, while HprK phosphorylates (and dephosphorylates) PtsH at the serine‐46 (Galinier *et al*., 1998; Dossonnet *et al*., 2000). To evaluate if His‐15 and Ser‐46 are also important active sites for the PtsH product in *Xcc* we carried out a set of alanine substitutions (see Methods). Here point mutants were generated in *ptsH*, where the His‐15 and Ser‐46 were replaced by Ala in the coding sequence and cloned into the plasmid pLAFR3 (see Methods). The generated constructs were named pLCptsH_H15A_ and pLCptsH_S46A_. These constructs were introduced into the double deletion mutant ΔhprK_Xcc_ΔptsH and the resulting strains was tested for various phenotypes. When pLCptsH_S46A_ was introduced into ΔhprK_Xcc_ΔptsH no changes in phenotypes were seen, however, when pLCptsH_H15A_, was introduced into the *hprK*_*Xcc*_ */ptsH* double mutant the resulting strain displayed similar phenotypes to the *hprK*_*Xcc*_ single mutant (Supporting Information Fig. S4), implying Ser‐46 is required for PtsH regulatory activity. These combined data suggest that the *ptsH* gene has an epistatic relationship with the *hprK*_*Xcc*_ gene and that *ptsH* may function downstream of *hprK*_*Xcc*_ in a regulatory pathway.

### *Overexpression of PtsH influences the phenotypes regulated by HprK_Xcc_*

The observations described reveal that *ptsH* gene has an epistatic relationship with the *hprK*_*Xcc*_ gene and suggests that an elevated levels of PtsH contributes to the regulatory action HprK_Xcc_ in *Xcc*.

**Figure 6:**
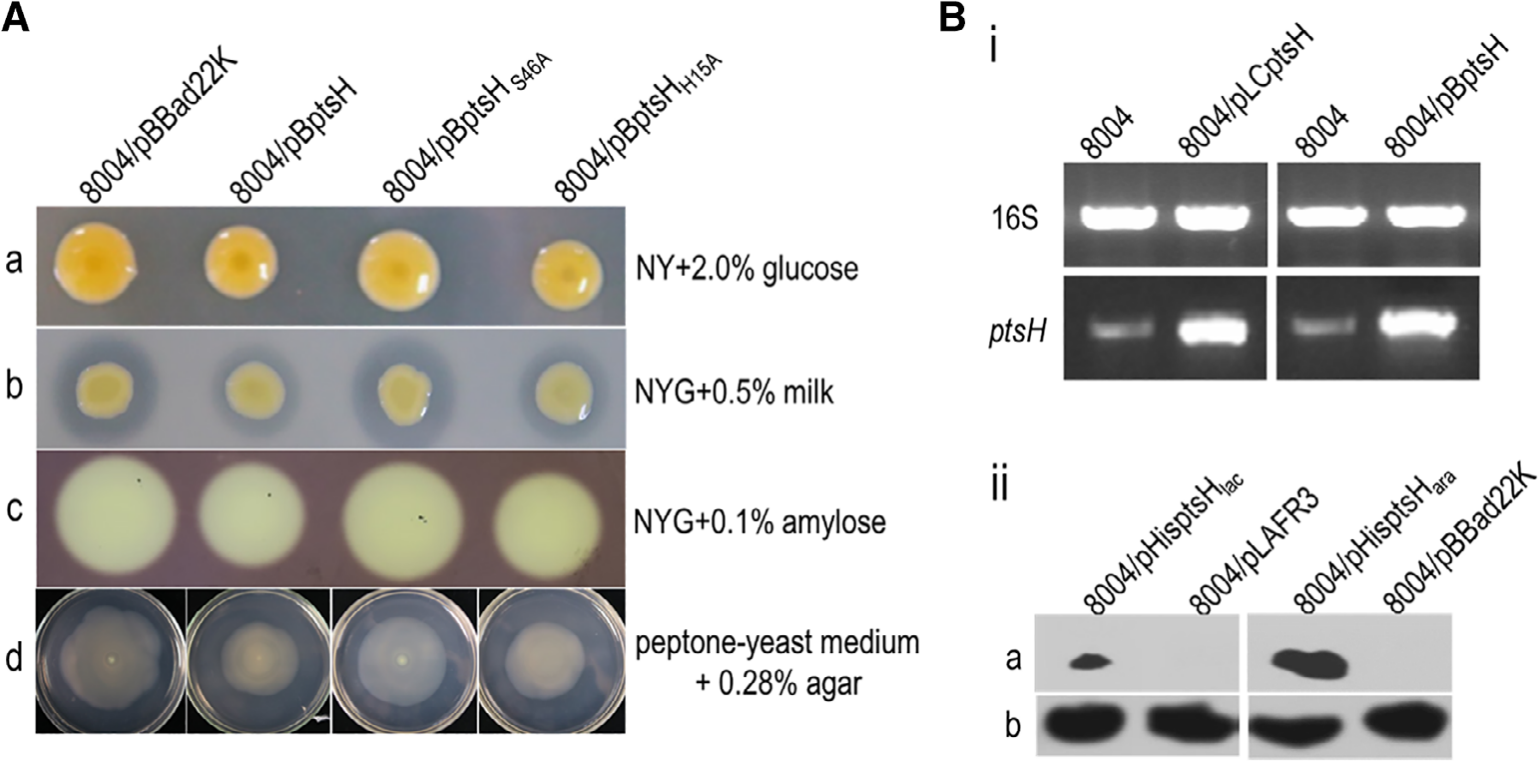
Overexpression of PtsH protein reduces the EPS production, activity of extracellular enzymes and cell motility in *Xcc*. A. Plate assays were used to test the EPS production (a), the activity of extracellular enzymes (b, c) and motility (d). Here an overnight culture (2 μl, OD_600_ = 1.0) of each *Xcc* strain was spotted onto a tested plate containing 0.2% (wt/vol) L‐arabinose. For EPS production, bacteria on NY plates containing 2.0% (wt/vol) glucose were incubated at 28°C for 5 days. The strain 8004/pBptsH displayed small colonies to the control strain 8004/pBBad22K, indicating the EPS yield of 8004/pBptsH strain was less compared to that of the control strain. For estimation of the activity of extracellular enzymes, strains on NYG plates 0.5% (wt/vol) skim milk (for protease) or 0.25% (wt/vol) carboxymethylcellulose (for endoglucanase) were incubated at 28°C for 24 h (endoglucanase) or 48 h (protease). Zones of clearance around the spot, which due to the degradation of the substrate, from strain 8004/pBptsH were small compared to the control strain, indicating the activity of extracellular enzymes of strain 8004/pBptsH was less to that of the wild type. Similar results were obtained in two other independent experiments. To detect swimming motility, an overnight culture (OD_600_ of 1.0) of each *Xcc* strain was stabbed into 0.28% agar plates composed of 0.03% Bacto peptone and 0.03% yeast extract followed by incubation at 28°C for 4 days. B. *ptsH* driven by arabinose‐inducible (*ara*) promoter in *Xcc* produces high concentration of PtsH protein. (i) Reverse‐transcription PCR (RT‐PCR) assay to examine the transcription level of *ptsH* gene in *Xcc* strains. RT‐PCR was performed using the synthesized cDNAs from the extracted total RNAs of the *Xcc* strains grown in NY medium for 20 h as templates to amplify the internal sequence of *ptsH* gene with primer set 1305NF/R. PCR fragment of *ptsH* from strain 8004/pBptsH was diluted in 10 times before electrophoresis analysis. The 16S rRNA gene in *Xcc* strains was used as a control. (ii) Western blot assay to examine the translation level of PtsH protein in *Xcc* strains. The recombinant plasmids pHisptsH_lac_ and pHisptsH_ara_, which contains the PtsH coding sequence fused with 6 × His tag in its C‐terminus, were introduced into *Xcc* strain 8004. The resulting recombinant strains were cultured in NYG medium with (for strain 8004/pHisptsH_ara_) or without (for strain 8004/pHisptsH_lac_) L‐arabinose for 12 h, and the total proteins in *Xcc* cells were prepared as previously described (Zang *et al*., 2007). Thirty micrograms of cell protein was electrophoresed in SDS‐PAGE gel and transferred to a PVDF membrane. The presence of PtsH protein subject to *lac* or *ara* promoter was detected by anti‐6 × His monoclonal antibody (a). As a loading reference, the blot was also probed with an anti‐RNA polymerase β‐antibody (b). [Color figure can be viewed at http://wileyonlinelibrary.com]

To validate this assumption, the recombinant plasmid pLCptsH (plasmid pLAFR3 harbouring 270‐bp *ptsH* ORF) was introduced into the wild‐type strain 8004, resulting strain 8004/pLCptsH. Interestingly, the strain 8004/pLCptsH showed wild type phenotypes (data not shown). This might due to the expression level of the *ptsH* is not elevated effectively, or the proportion of phosphorylated PtsH is inappropriate. The 270‐bp *ptsH* gene was therefore amplified from *Xcc* genomic DNA by PCR and cloned into pBBad22K, under the control of an arabinose‐inducible promoter generating the construct pBptsH (see Methods) (Sukchawalit *et al*., 1999). The pBptsH construct was introduced into *Xcc* wild‐type strain 8004 using triparental conjugation, resulting in strain 8004/pBptsH. As a control, vector pBBad22K was also introduced into wild‐type strain 8004. These strains were tested for changes in extracellular enzymes (protease & amylase), EPS production, and cell motility in the presence of 0.2% (wt/vol) L‐arabinose to induce the expression of *ptsH* (see Methods). As showed in Fig. 6A, 8004 strain harbouring recombinant plasmid pBptsH showed similar phenotypes to the *hprK*_*Xcc*_ mutant. Furthermore, the *ptsH* gene that carried variant code for His‐15 and Ser‐46 (replaced by alanine) were also cloned into the plasmid pBBad22K, the resulting plasmids pBptsH_H15A_ and pBptsH_S46A_ were introduced into *Xcc* wild‐type strain 8004 (see Methods). Phenotype analysis revealed that, strain 8004 containing pBptsH_H15A_ but not pBptsH_S46A_ present altered phenotypes, indicating overproducing PtsH with Ser‐46 replacement had no impact on the phenotypes, and Ser‐46 is essential for the regulatory function of PtsH protein in *Xcc*.

In parallel, we evaluated the expression of *ptsH* in wild‐type 8004 strain containing recombinant plasmid pBptsH using RT‐PCR. *Xcc* strains wild‐type 8004, 8004/pLCptsH and 8004/pBptsH were assessed from *ptsH* gene expression (see Methods). Results revealed that the band representing *ptsH* fragments from strain 8004/pBptsH was more obvious than that from 8004 strain indicating the transcription level of *ptsH* was effectively elevated in strain 8004/pBptsH (Fig. 6B‐i). Although the expression data revealed that the transcription level of *ptsH* was elevated in strain 8004/pBptsH, western blotting was further performed to confirm that *Xcc* strains produces high concentration of PtsH (HPr) protein. To do this, the recombinant plasmid pHisptsH_lac_ and pHisptsH_ara_, were introduced into *Xcc* wild‐type strain 8004 (see Methods). The resulting strains 8004/pHisptsH_lac_ and 8004/pHisptsH_ara_, as well as the wild‐type strain 8004 were used to examine the fusing protein 6 × His‐PtsH. As shown in Fig. 6B‐ii, the band representing the fusion protein 6 × His‐PtsH present in the strain 8004/pHisptsH_lac_ and 8004/pHisptsH_ara_. Moreover, the band from strain 8004/pHisptsH_ara_ was more intense than that from strain 8004/pHisptsH_lac_. These data indicated that 8004 strain harbouring plasmid pHisptsH_ara_ is able to produce a higher concentration of PtsH protein.

### *HprK_Xcc_ is a functional serine kinase*

HprK from Gram‐positive bacteria have been shown to exert their regulatory action by catalysing the phosphorylation of the serine (or threonine) residue at position 46 of the PtsH protein (Galinier *et al*., 1998; Dossonnet *et al*., 2000). In order to investigate if HprK_Xcc_ has similar biochemical activity, we purified the protein and tested its ability to phosphorylate PtsH.

**Figure 7:**
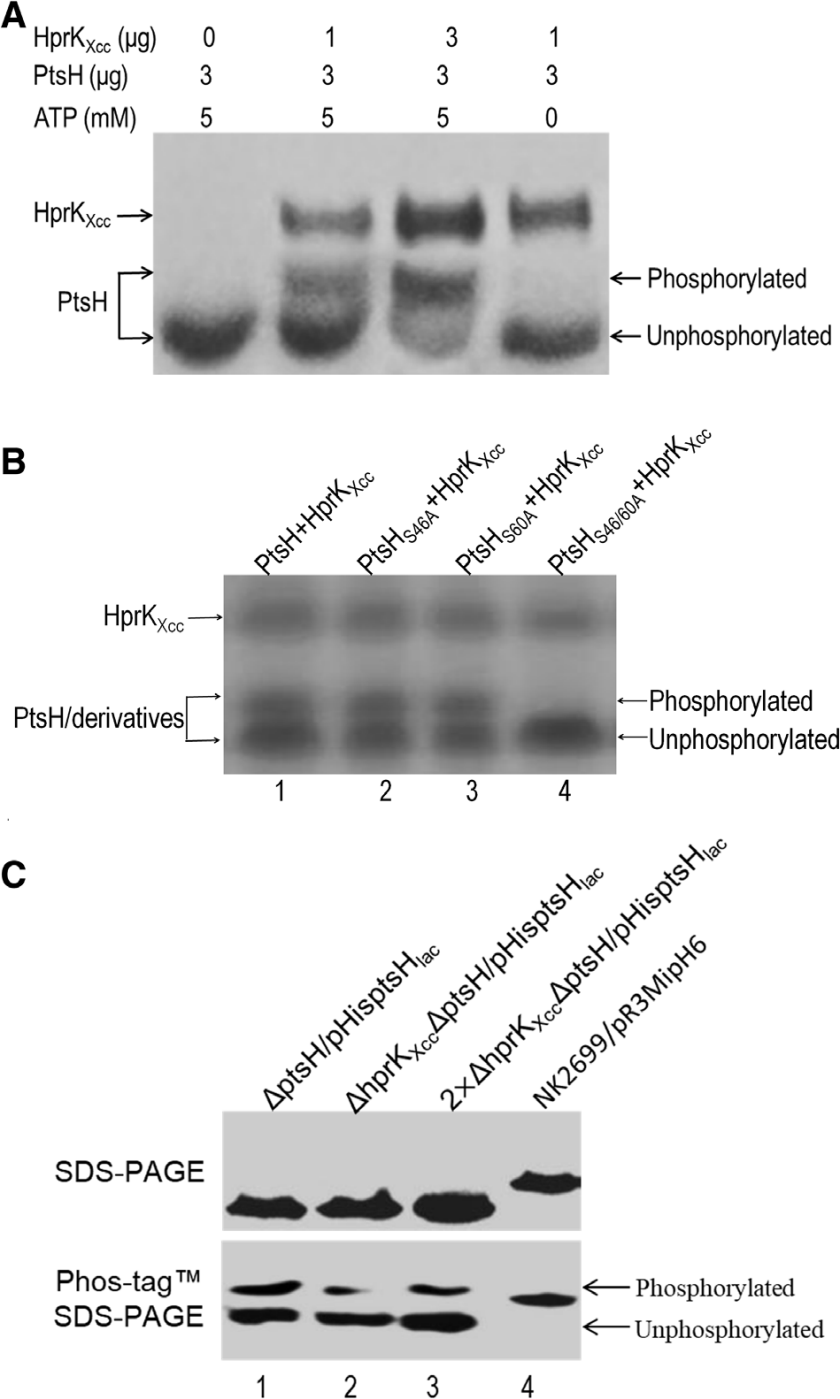
HprK_Xcc_ is an active serine protein kinase *in vitro* and *in vivo*. A. *In vitro* phosphorylation of PtsH protein by HprK_Xcc_ of *Xcc*. The 50 μl reaction mixtures containing a variety amounts of ATP, PtsH and HprK_Xcc_ (indicated above the figure) were incubated at 25°C for 30 min. After the reactions stopped, the phosphorylated and unphosphorylated forms of PtsH protein were separated by electrophoresis using Phos‐tagTM SDS‐PAGE gel. B. Phosphorylation test of the PtsH derivatives by HprK_Xcc_. The phosphorylation reaction was conducted in 50 μl volume containing 3 μg PtsH protein or its derivatives, 1 μg HprK_Xcc_ and 5 mM ATP (The proteins of each lane is shown above the figure). S46A mutant PtsH (lane 2) and S60A mutant PtsH (lane 3) but not the S46/60A mutant PtsA (lane 4) can be phosphorylated by HprK_Xcc_. C. HprK_Xcc_ has an impact on the phosphorylation of wild‐type PtsH protein *in vivo*. *Xcc* strains expressing PtsH protein (or its derivatives) with a 6 × His‐tag were cultured in NYG medium and total proteins were prepared. About 10 or 20 μg (for 2 × ΔhprK_Xcc_ΔptsH/pHisptsHlac) of samples were separated on SDS‐PAGE and Phos‐tag™ SDS‐PAGE gels respectively, and then electro‐transferred onto PVDF membrane for Western blotting. The primary antibody was anti‐His‐tag antibody (Qiagen) that was used at a 1:2500 dilution according to manufacturer's instructions. Binding of the primary antibody was detected using goat anti‐mouse IgG horseradish peroxidase conjugated secondary antibody (Bio‐Rad). Phosphorylated and unphosphorylated PtsH proteins were separated by Phos‐tag SDS‐PAGE gel. Protein sample from strain NK2699/pR3MipH6 expressing Mip protein with a 6 × His‐tag was used as a negative control.

For these experiments HprK_Xcc_ was 6 × His‐tagged and cloned into *Escherichia coli* strain M15 for overexpression and purification of the protein (see Methods). PtsH was also purified where the 270‐bp *ptsH* gene was amplified and cloned into the expression vector pET‐32a, and the resulting construct pET‐PtsH was transferred into *E. coli* strain BL21 (DE3) (see Methods). After purification, the thioredoxin domain, as well as the His‐ and S‐tag, was cleaved from the fusion protein using enterokinase. The ATP‐dependent phosphorylation assays were carried out with purified PtsH protein and 6 × His‐HprK_Xcc_ fusion protein (see Methods). As shown in Fig. 7A, in the absence of 6 × His‐HprK_Xcc_ protein no phosphorylated band was generated, however, with increasing amounts of 6 × His‐HprK_Xcc_ protein it appears that phosphorylated PtsH protein band grows more intense, indicating that *Xcc* HprK_Xcc_ protein has kinase activity.

To extend this study and test if HprK_Xcc_ phosphorylation of PtsH protein is dependent on the serine residue at position 46 we generated a PtsH where Ser‐46 was replaced with Ala‐46 (see Methods). The obtained PtsH_S46A_ protein was examined using the ATP‐dependent phosphorylation assays. The results revealed that the PtsH_S46A_ was still phosphorylated by HprK_Xcc_ (Fig. 7B), indicating another residue may be phosphorylated. Examination of the PtsH protein sequence from *Xcc* revealed that another serine residue present at the position 60 is another candidate. We generated other variant PtsH proteins where alanine substitutes serine at position 60 (PtsH_S60A_) and positions 46 and 60 (PtsH_S46A/S60A_). The ATP‐dependent phosphorylation test with these variant PtsH proteins was then carried out. As shown in Fig. 7B, a phosphorylated band was present with the mutant protein PtsH_S46A_ and PtsH_S60A_ but not PtsH_S46/60A_, indicating that the two serine residues at position 46 and 60 could be phosphorylated by HprK_Xcc_, and that the serine residue at position 60 may be an alternate phosphorylation site in *Xcc* PtsH protein.

As all the experiments described above examined the phosphorylation PtsH by HprK_Xcc_ using protein expressed and purified from *E. coli* strains (*in vitro*), we wished to evaluate the impact of HprK_Xcc_ on the PtsH phosphorylation in the *Xcc* cell. To achieve this the level of phosphorylation of PtsH within the *Xcc* backgrounds were compared using a Phos‐tag™ SDS‐PAGE method in tandem with western blotting. To do this, the recombinant plasmid pHisptsH_lac_ expressing PtsH protein with a 6 × His‐tag was introduced into the ΔptsH and ΔhprK_Xcc_ΔptsH strains. The resulting strains ΔptsH/pHisptsH_lac_ and ΔhprK_Xcc_ΔptsH/pHisptsH_lac_ were cultured in NYG medium, and total protein was prepared from the bacterial cells. After fractionation using Phos‐tag™ SDS‐PAGE and SDS‐PAGE gels, PtsH protein was detected using western blotting (see Methods). As shown in Fig. 7C, when the same amount of total protein was loaded (lane1, 2), the bands representing total PtsH protein from the tested strains was similar, however, the bands representing the phosphorylated PtsH protein from strain ΔhprK_Xcc_ΔptsH*/*pHisptsH_lac_ were faint compared to that from strain ΔptsH/pHisptsH_lac_. The band representing the phosphorylated PtsH protein from ΔptsH/pHisptsH_lac_ total protein was greater than that from 2‐times ΔhprK_Xcc_ΔptsH/pHisptsH_lac_ total protein (lane 3), suggesting that the amount of phosphorylated PtsH protein in wild‐type background was greater than that in *hprK*_*Xc*c_‐mutant background (Fig. 7C).

## Discussion

**Figure 8:**
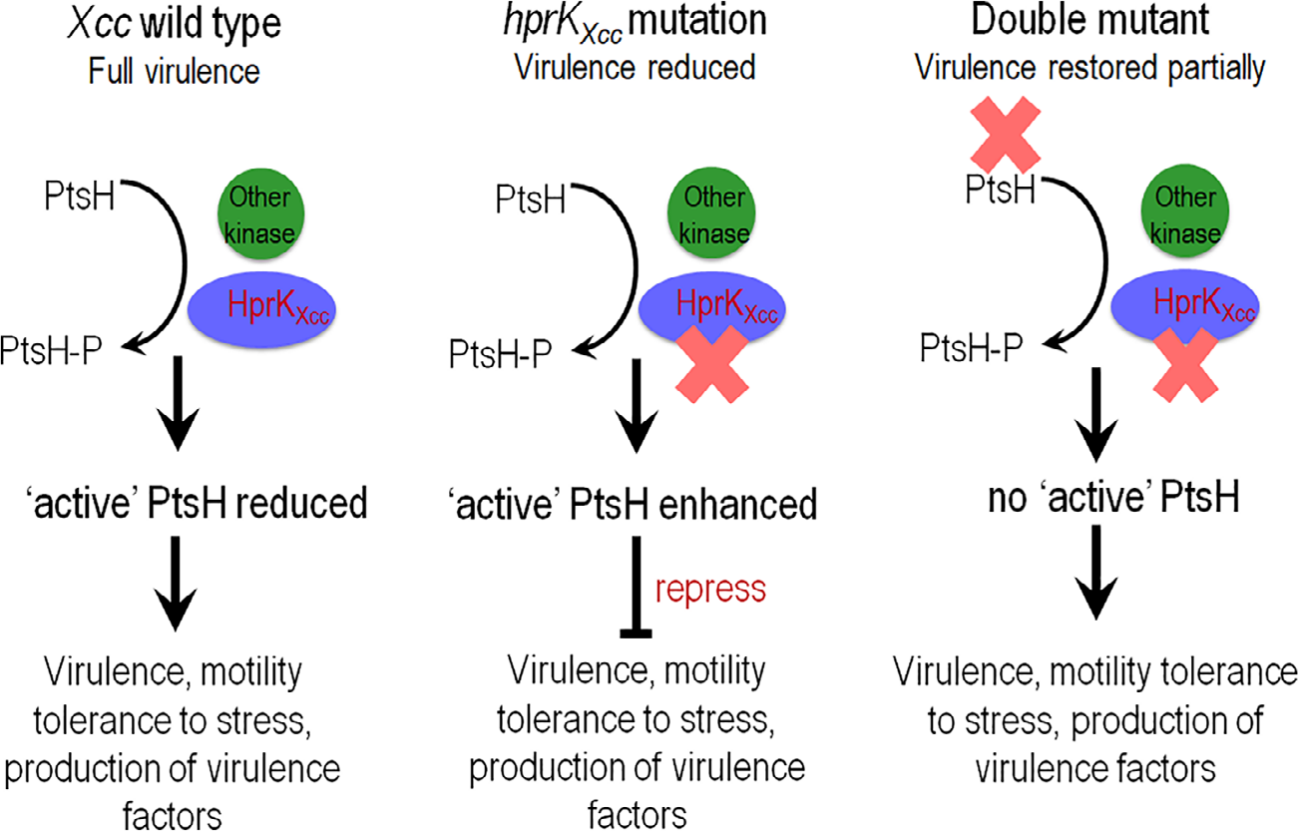
Model for the regulatory action of HprK_Xcc_ and PtsH in *Xcc*. HprK_Xcc_ responds to unknown environmental signals. HprK_Xcc_ appears to be one of a number of kinase proteins involved in the phosphorylations/dephosphorylation the protein PtsH. HprK_Xcc_ appears to reduce the regulatory activity of PtsH protein by modulation its phosphorylation state *via* the serine‐46 residue. ‘Active’ PtsH protein negative regulates biological processes including virulence factor synthesis in *Xcc*. [Color figure can be viewed at http://wileyonlinelibrary.com]

It is now appreciated that many bacteria do not encode a complete PTS but these partial systems are believed to be retained as they play key roles in regulation of various biological processes (Reizer *et al*., 1998; Hu and Saier, 2002; Stonestrom *et al*., 2005). These partial PTS are commonly encoded in the Gram‐negative but few contain genes encoding HprK proteins seen in most Gram‐positive bacteria (Reizer *et al*., 1998; Hu and Saier, 2002; Stonestrom *et al*., 2005). Here, we show a partial PTS is encoded in the genome of the plant pathogen *Xcc* that includes the general proteins Enzyme I (PtsI, XC_1304), HPr (PtsH, XC_1305) and two EIIA‐like proteins EIIA^Man^ (PtsN^Man^, XC_1306) and EIIA^Ntr^ (PtsN^Ntr^, XC_1309). However, the other transport‐related PTS proteins like CcpA, EIIB and EIIC do not appear to be present. Our functional assessment revealed that these components played no major role in sugar transport. Although, further functional tests revealed that HprK_Xcc_ (but not the other components) were required for the regulation of virulence associated traits including extracellular enzyme activity, extracellular‐polysaccharide production, cell motility and the full virulence of *Xcc* to Chinese radish. Additional HprK_Xcc_, transcription analysis revealed that this protein has a global regulatory role controlling the expression of over 250 genes in the *Xcc* genome under the conditions tested. Moreover, through overexpression and gene deletion analysis we demonstrate that the gene *hprK*_*Xcc*_ shares an epistatic relationship with *ptsH*. Additionally, our biochemical tests showed that HprK_Xcc_ is a functional serine kinase, which has the ability to phosphorylate PtsH. These results illustrate a complex regulatory mechanism in *Xcc* by previously uncharacterized PTS components and underscore the importance of HprK_Xcc_ in the regulation of virulence in this important plant pathogen. Furthermore, to our knowledge, this is the first report of an HprK (Ser) kinase showing global control of virulence associated functions in a Gram‐negative bacterium (Fig. 8).

In Gram‐positive bacteria, HprK has been shown to regulate catabolite repression and sugar transport by phosphorylating/dephosphorylating the PTS protein PtsH on a conserved serine residue at position 46, resulting seryl‐phosphorylated PtsH. P‐Ser‐PtsH (also named P‐Ser‐HPr) regulates carbohydrate metabolism via forming a complex with CcpA (catabolite control protein A). It was believed that HprK was confined to Gram‐positive bacteria but recent bioinformatics analysis of genome sequences has revealed that some Gram‐negative bacteria also possess HprK homologues and component of the PTS although these are usually incomplete (Reizer *et al*., 1998; Hu and Saier, 2002; Stonestrom *et al*., 2005). Biochemical studies have shown that these HprK or other PTS components have similar activity to their Gram‐negative counterparts but the physiological functions they are involved in are less well‐defined. In this work, we showed that HprK_Xcc_ is functional in an ATP‐dependent phosphorylation assay using purified HprK_Xcc_(6 × His) and PtsH. This was further confirmed by Phos‐tag™ SDS‐PAGE method where HprK_Xcc_ was shown to phosphorylate PtsH in the *Xcc* cell. Further analysis revealed that PtsH carrying single variants PtsH_S46A_ or PtsH_S60A_ were phosphorylated by HprK_Xcc_ *in vitro*_._ In contrast, the HprK_Xcc_ failed to phosphorylate a PtsH protein which carried two alanine substitutes in the two conserved serine residues at positions 46 and 60 (PtsH_S46A/S60A_). Unlike HprK proteins from other bacteria the phosphorylation of PtsH protein was not dependant on a single serine but rather both conserved serine residues 46 or 60 i*n vitro*. The reason for this is still enigmatic.

We have provided evidence consistent with a model wherein HprK_Xcc_ phosphorylates PtsH at the residues serine‐46, an event that is important in the regulation of virulence in *Xcc* (Fig. 8). The PtsH protein in Gram‐positive bacteria is also phosphorylated by the Enzyme I of the PTS at the residues histidine‐15, resulting histidyl‐phosphorylated PtsH (P‐His‐PtsH) (Deutscher *et al*., 2014). PtsH not only acts as a phosphoryl carrier within the PTS phosphorylation cascade but also the key regulator of carbon metabolism. It performs diverse regulatory functions based on its phosphorylation state (Görke & Stülke, 2008). The physiological function of the PtsH homologues in Gram‐negative still unknown. Here, the *ptsH* deletion mutant present the wild type phenotypes, indicating that the absence of PtsH/or P‐Ser‐PtsH/or cerain conformation of PtsH has no impact on the virulence and other phenotypes in *Xcc*. However, the double deletion mutant of HprK_Xcc_/PtsH present similar wild‐type phenotypes, and the PtsH derivative in which Serine‐46 was substituted by alanine cannot complement the *hprK*_*Xcc*_/*ptsH* double deletion mutant, suggesting the Serine‐46 in PtsH is essential for the PtsH regulatory function. We therefore supposed that a certain concentration of PtsH or proportion of phosphorylated PtsH (also named ‘Active’ PtsH), which might be reduced by the phosphorylation on Serine‐46 by HprK_Xcc_, is responsible for regulatory function in series phenotypes in *Xcc* (Fig. 8). To ascertain this hypothesis, *ptsH* and its derivates were overexpressed in the wild‐type strain 8004 respectively. Consistence with our assumption, the overproduction of PtsH and the mutant form on Histidine‐15 (PtsH_H15A_), but not the mutant form on Serine‐46, in *Xcc* wild‐type strain 8004 alter the phenotypes of this bacterium. These combined data indicated that the regulation mechanism of HprK_Xcc_ and PtsH proteins in *Xcc* differ from those in Gram‐positive bacteria. HprK_Xcc_ might reduce the regulatory activity of PtsH protein *via* phosphorylated on its Serine‐46, and the ‘Active’ PtsH protein most likely affects a variety of biological processes *via* interactions with target protein or/and DNA (Fig. 8).

HprK_Xcc_ indirectly or directly regulates virulence gene transcription and phenotypes associated with virulence in *Xcc*. Despite these observations additional studies are needed to examine the role of HprK_Xcc_ in the regulation of phenotypes discovered but also many other questions could be addressed: What are the environmental cues that activate the expression and activity of HprK_Xcc_? What are the specifics of HprK_Xcc_ phosphorylation PtsH, can we gain structural insight? Does HprK_Xcc_ phosphorylate other target proteins? How does HprK_Xcc_ modulate gene expression in *Xcc*? Overall, this work illustrates the previously unappreciated global regulatory role of HprK_Xcc_ and previously uncharacterized PTS components that control of virulence in this Gram‐negative bacterial plant pathogen.

## Materials and methods

### *Bacterial strains, plasmids and growth conditions*

The bacterial strains and plasmids used in this study are listed in Supporting Information Table S1. The *E. coli* strains were grown in Luria‐Bertani medium (Miller, 1972) at 37°C. The *Xcc* strains were grown at 28°C in NYG medium (Daniels *et al*., 1984a) or NY medium (NYG medium but without glycerol), the minimal medium MMX (Daniels *et al*., 1984b), NCM (MMX medium without citric acid and glucose). Antibiotics were added at the following concentrations as required: kanamycin (Kan) at 25 μg ml^−1^; rifampicin (Rif) at 50 μg ml^−1^; ampicillin (Amp) at 100 μg ml^−1^; spectinomycin (Spc) at 50 μg ml^−1^ and tetracycline (Tet) at 5 μg ml^−1^ for *Xcc* and 15 μg ml^−1^ for *E. coli*.

### *DNA and RNA manipulations*

The DNA manipulations followed the procedures described by Sambrook *et al*. (1989). Conjugation between the *Xcc* and *E. coli* strains was performed as described by Turner *et al*. (1985). The restriction endonucleases, T4 DNA ligase and *pfu* polymerase were provided by Promega (Shanghai, China). The total RNAs were extracted from the cultures of the *Xcc* strains with a total‐RNA extraction kit (Invitrogen, Waltham, MA, USA) and cDNA generated using a cDNA synthesis kit (Invitrogen). For Semi‐quantitative reverse‐transcription PCR (RT‐PCR), the obtained cDNA was diluted and used as a template with selected primers (see Supporting Information Table S5) for target genes.

To assay the transcription level of certain genes (e.g*. gumB*, *xcsC*, *pelB*, *egl*), quantitative real‐time PCR (qRT‐PCR) was carried out as previously described (Li *et al*., 2014). The synergy brand (SYBR) green‐labelled PCR fragments were amplified using the corresponding primer set (Supporting Information Table S5). The relative mRNA level was calculated with respect to the level of the corresponding transcript in the wild‐type strain 8004 (equalling 1). The expression level of the 16S rRNA gene was used as an internal standard. The qRT‐PCR tests were performed in triplicate.

### *Deletion mutant construction and complementation*

Single‐deletion mutant of *ptsI* (*XC_1304*), *ptsH* (*XC_1305*), *ptsN* ^*Man*^ (*XC1306*), *ptsN* ^*Ntr*^ (*XC_1309*) or *hprK*_*Xcc*_ (*XC_1308*) was constructed using the method described by Schäfer *et al*. (1994). In general, 500–700 bp upstream and downstream fragments of the target gene were amplified using the corresponding primer set (Supporting Information Table S5). Primers were modified to give *Eco*RI, *Xba*I or *Hin*dIII‐compatible ends. The two fragments were cloned together into the vector pK18*mobsacB* (Schäfer *et al*., 1994), the resulting recombinant plasmid was introduced into *Xcc* strain 8004 by triparental conjugation, and transconjugants were screened on selective agar plates containing 5% sucrose. The obtained mutants of *ptsI*, *ptsH*, *ptsN* ^*Man*^, *ptsN* ^*Ntr*^ and *hprK*_*Xcc*_ were named ΔptsI, ΔptsH, ΔptsN^Man^, ΔptsN^Ntr^ and ΔhprK_Xcc_ respectively (Supporting Information Table S1).

For complementation of the *hprK*_*Xcc*_ single deletion mutant, a 1201‐bp DNA fragment containing the *hprK*_*Xcc*_ coding region and extending from 180 bp upstream of the 5′ end to 70 bp downstream of the 3′ end of the ORF was amplified by PCR from the total DNA of *Xcc* strain 8004 with the primer set ChprKF/R (Supporting Information Table S5). Primers were modified to give *Bam*H or *Hin*dIII‐compatible ends (underlined). The amplified fragment was confirmed by sequencing, and ligated into the *Bam*HI and *Hin*dIII sites of the plasmid pLAFR3 (Staskawicz *et al*., 1987), generating the recombinant plasmid pLChprK_Xcc_ (Supporting Information Table S1). The plasmid was introduced into the *hprK*_*Xcc*_ deletion mutant ΔhprK_Xcc_ by triparental conjugation, generating a complemented strain named CΔhprK_Xcc_ (Supporting Information Table S1). Simultaneously, the empty vector pLAFR3 was also introduced into ΔhpaS, resulting a strain used as a control.

To construct a double‐deletion mutant of *hprK*_*Xcc*_/*ptsI*, *hprK*_*Xcc*_/*ptsH*, *hprK*_*Xcc*_/*ptsN* ^*Man*^ or *hprK*_*Xcc*_/*ptsN* ^*Ntr*^, the method described by Schäfer *et al*. (1994) was employed. Upstream and a downstream fragments of *ptsI*, *ptsH*, *ptsN* ^*Man*^ or *ptsN* ^*Ntr*^ were cloned together into the vector pK*18mobsacB* (Schäfer *et al*., 1994), and the resulted plasmid was introduced into the *hprK*_*Xcc*_ single deletion mutant ΔhprK_Xcc_ by triparental conjugation. The transconjugants were screened on selective agar plates containing 5% sucrose. The obtained double deletion mutant was further confirmed by PCR and named ΔhprK_Xcc_ΔptsI, ΔhprK_Xcc_ΔptsH, ΔhprK_Xcc_ΔptsN^Man^ and ΔhprK_Xcc_ΔptsN^Ntr^ respectively.

For complementation of *hprK*_*Xcc*_/*ptsH* double deletion mutant, a 270‐bp DNA fragment of the *ptsH* ORF sequence was amplified by PCR using the primer set CptsH‐F/R (Supporting Information Table S5) and cloned into *Bam*HI/*Hin*dIII sites of the plasmid pLAFR3, resulting plasmid pLCptsH (Supporting Information Table S1). This recombinant plasmid was introduced into the *hprK*_*Xcc*_ and *ptsH* double deletion mutant ΔhprK_Xcc_ΔptsH, the obtained complemented strain was named ΔhprK_Xcc_ΔptsH/pLCptsH (Supporting Information Table S1).

For overexpression of *ptsH* in *Xcc*, the 270‐bp DNA fragment of *ptsH* coding sequence amplified using the primer set ptsH‐1F/R (Supporting Information Table S5) was cloned into *Kpn*I/*Xba*I sites of the broad‐host‐range expression vector pBBad22K (Sukchawalit *et al*., 1999), obtaining recombinant plasmid pBptsH. This recombinant plasmid was introduced into the *Xcc* wild‐type strain 8004, resulting strain 8004/pBptsH (Supporting Information Table S1).

### *Site‐directed mutagenesis*

For site‐directed mutagenesis of *ptsH* was performed with a QuikChange II Site‐directed Mutagenesis kit (Stratagene, La Jolla, CA, USA) using the primer sets HptsH‐F/R and SptsH‐F/R and recombinant plasmid pKptsH as template (Supporting Information Table S5). Here amino acid (aa) substitutions of histidine residue at position 15, serine residue at position 46, serine residue at position 60, both the position 15 and 46, and both the position 46 and 60 in PtsH product were developed. pKptsH was derived from the above 270‐bp DNA fragment of *ptsH* ORF cloned into the suicide plasmid pK18*mob* (Schäfer *et al*., 1994). Final constructs were digested with *Bam*HI/*Hin*dIII, and the mutated 270‐bp DNA fragments were cloned into plasmid pLAFR3, resulted recombinant plasmid pLCptsH_H15A_, pLCptsH_S46A_ and pLCptsH_15/46A_ (Supporting Information Table S1) respectively. The obtained recombinant plasmids were used for complementation test.

### *Protein overproduction and purification*

To overproduce 6× His‐tagged HprK_Xcc_ protein, a 948‐bp of *hprK*_*Xcc*_ coding sequence of *Xcc* strain 8004 was PCR‐amplified using primer set OhprK‐F/R and cloned into the expression plasmid pQE‐30, resulting recombinant plasmid pQE‐HprK_Xcc_. The recombinant plasmid was then transformed into *E. coli* strain M15, resulting strain M15/pQE‐HprK_Xcc_. After cultivation and induction by IPTG (isopropyl‐thiogalactopyranoside), the cells were harvested and 6 × His‐tagged fused proteins 6 × His‐HprK_Xcc_ were purified by Nickel‐NTA resin (Qiagen, Hilden, Germany).

For overproduction of *Xcc* PtsH protein (or the mutant form protein), a 270‐bp ORF sequence of *ptsH* (or the point mutated *ptsH* gene) was PCR‐amplified using primer set CptsH‐F/R, and the obtaining DNA fragments were coloned into the expression vector pET‐32a, the resulting recombinant plasmid pET‐PtsH (or pET‐PtsH_S46A_, pET‐PtsH_S60A_, pET‐PtsH_S46/60A_, pET‐PtsH_S46/60T_) was transformed into *E. coli* strain BL21 (DE3), resulting a recombinant strain BL21/pET‐ PtsH (or BL21/pET‐PtsH_S46A_, BL21/pET‐PtsH_S46/60A_, BL21/pET‐PtsH_S46/60T_) producing a thioredoxin‐PtsH fusion protein with His and S tags located between the fused proteins. After overproduction and purification, the purified fusion protein was treated with enterokinase to cut off the thioredoxin domain and the His‐ and S‐tag, and PtsH (or PtsH_S46A_, PtsH_S60A_, PtsH_S46/60A_, PtsH_S46/60T_) protein was purified on a Nickel‐NTA resin. The concentration of the purified protein was determined by Bradford assay (Bradford, 1976).

### In vitro *phosphorylation assay*

For the ATP‐dependent phosphorylation of PtsH (or PtsH mutant forms PtsH_S46A_, PtsH_S60A_ and PtsH_S46/60A_) protein with HprK_Xcc_, the 50 μl reaction mixtures contained: 30 mM Tris–HCl, pH 8.0; 50 mM KCl，10 mM MgCl_2_，5 mM ATP, 3 μg PtsH and varing amounts of 6 × His‐HprK_Xcc_. After incubation at 25°C for 30 min, the reactions were stopped by heating for 5 min at 75°C. The phosphorylated and unphosphorylated forms of PtsH protein were separated by electrophoresis using Phos‐tag™ SDS‐PAGE gel.

### In vivo *phosphorylation assay and western blotting*

Phosphorylation of PtsH protein (encoded by *ptsH*) *in vivo* was analysed by using Phos‐tag™ SDS/PAGE combined with western blotting as previous described (Li *et al*., 2014). *Xcc* strains expressing PtsH protein with a 6 × His‐tag on its C‐terminus were first constructed. A 288‐bp DNA fragment containing a promoterless *ptsH* ORF fused with a 6 × His‐tag encoding sequence was PCR‐amplified using the primer set ptsH‐2F/R (Supporting Information Table S5). The obtained DNA fragment was cloned into the vector pLAFR3 in an orientation that allowed the *ptsH* to be driven by the *lac* promoter. The obtained recombinant plasmid pHisptsH_lac_ was introduced into the *ptsH* deletion mutant strain ΔptsH and *hprK*_*Xcc*_/*ptsH* double‐deletion mutant strain ΔhprK_Xcc_ΔptsH respectively. The resulting strains ΔptsH/pHisptsH_lac_ and ΔhprK_Xcc_ΔptsH/pHisptsH_lac_ cultivated in NYG medium for 16 h, and total proteins from the bacterial cells were prepared.

Fifty micrograms of total protein of each sample was loaded per well in a Phos‐tag™ SDS/PAGE gel (Wako Pure Chemical Industries, Ltd, Osaka, Japan), and electrophoresis was performed. Simultaneously, samples were loaded onto a SDS‐PAGE gel and electrophoresed. Proteins were electrotransferred onto a PVDF (polyvinylidene difluoride) membrane (Millipore, Billerica, MA, USA). The membrane was subjected to western blot analysis using 1:2500 diluted anti‐His‐tag mouse monoclonal antibody (Qiagen, Shanghai, China) as a primary antibody. The diluted1:2500 horseradish peroxidase conjugated goat anti mouse IgG (Bio‐Rad, Hercules, CA, USA) was used as secondary antibody. Antibody reactions were visualized by chemiluminescence, which was performed according to the manufacturer's instructions.

### *Extracellular enzyme and xanthan gum assays*

The activity of extracellular enzymes was tested using a radial diffusion assay as previous described (Tang *et al*., 1991). To estimate quantitatively the activity of the extracellular enzymes endoglucanase (cellulase), amylase, pectate lyase and protease, *Xcc* strains were cultured in NYG medium for 12 h and adjusted to the same concentration, and then cells were removed from the medium by centrifugation and the supernatant was taken for assays. For endoglucanase, 10 μl of enzyme‐containing extracts was added to 200 μl of indicator buffer containing 1% (wt/vol) carboxymethylcellulose (CMC, Sangon, Shangshai, China) as the substrate. The reactions were carried out for 30 min at 28°C. The released reducing sugars were measured as D‐glucose equivalents, as described by Miller (1959). One unit (U) of the endoglucanase activity was defined as the amount of enzyme releasing 1 μmol of reducing sugar per minute. Amylase activity quantification was conducted in the same way as for the endoglucanase measurement, except that the substrate 1% (wt/vol) CMC was replaced by 1% (wt/vol) starch solution. For pectate lyase, the activity was determined by measuring the increase in the absorbance at 235 nm of polygalacturonic acid (PGA) using a modification of the method described by Collmer *et al*. (1988), whereby 100 μl of enzyme‐containing extracts in 100 mM Tris–HCl (pH 9.0) containing 500 μM CaCl_2_ and 0.2% (wt/vol) PGA were incubated at 30°C for 30 min. The reaction was stopped by the addition of 20 μl of 0.35 M HCl. One unit of pectate lyase activity was defined as the amount of enzyme that produced 1 μmol of unsaturated galacturonide per minute. For extracellular protease activity, the method described by Swift *et al*. (1999) was used.

To evaluate the EPS production, *Xcc* strains were grown on NYG agar plates supplied with 2% (wt/vol) glucose at 28°C for 5 days, and the *Xcc* colony sizes were compared. To quantitative EPS yield, *Xcc* strains were cultured in 100 ml NY liquid medium containing 2% (wt/vol) glucose at 28°C with shaking at 200 r.p.m for 3 days. EPS was precipitated from the culture supernatant with ethanol, then dried and weighed, as described by Tang *et al*. (1991).

### *Motility assays*

To test swimming motility, an overnight culture (OD_600_ of 1.0) of each *Xcc* strain was stabbed into 0.28% agar plates composed of 0.03% Bacto peptone and 0.03% yeast extract (peptone‐yeast medium) followed by incubation at 28°C for 4 days. To detect swarming motility, the bacterial cells were inoculated onto NY plates containing 2% glucose and 0.6% agar using a toothpick, and then incubated at 28°C for 3 days. The diameters of the area occupied by the bacterial cells were measured and these values were used to indicate the motility of the *Xcc* strains. The experiment was repeated at least three times.

### *Stress tolerance assay*

The well‐established and widely used minimal inhibitory concentration (MIC) method (Wiegand *et al*., 2008) was employed to test the resistance of the *Xcc* strains to several environmental stresses, including osmotic challenge (NaCl), sodium dodecyl sulphate (SDS), the organic solvent phenol and heavy metal salt (CdCl_2_) stress. Briefly, *Xcc* strains were cultured to an OD_600_ of 0.6 and diluted; then 100 μl of the diluted culture was plated on NYG plates supplemented with different concentrations of each reagent respectively. The surviving colonies on the plates were counted after 3 days of incubation at 28°C.

### *Plant assay*

The virulence of *Xcc* to Chinese radish (*Raphanus sativus*) was tested by the leaf‐clipping method (Dow *et al*., 2003; Ryan *et al*., 2007). Leaves were cut with scissors dipped in the bacterial suspensions of an OD_600_ of 0.01 (1 × 10^7^ CFU ml^−1^). Lesion length was measured 10 days after inoculation, and data were analysed by *t*‐test. The growth of bacteria in radish leaf tissue was measured by homogenizing a group of leaves (five leaves for each sample) in 9 ml sterile water. Diluted homogenates were plated on NYG agar plates supplemented with corresponding antibiotics, and bacterial CFU were counted after incubation for 3 days.

### *Transcriptome analysis*

Transcriptome analysis were performed as previously described (Cui *et al*., 2018). In brief, RNAs were extracted from *Xcc* strains cultured in NYG medium to an OD_600_ of 0.6. Contaminated genomic DNA was removed with RNase‐free DNase I and verified by PCR. After RNA quantity determination and RNA quality assessment, total RNA was sent to Novogene‐Beijing for further treatments, library construction and strand‐specific RNA sequencing. Sequencing libraries were generated using a NEBNext Ultra™ Directional RNA Library Prep Kit for Illumina (New England BioLabs), and sequenced on an Illumina (CA, USA) HiSeq 2000 platform. Clean reads were mapped to the reference genome and the RPKM (reads per kilobase per million mapped reads) method was used to calculate the gene expression levels. False discovery rate FDR ≤0.05 and |log_2_FC| (log_2_ of the fold changes) ≥1 were considered for differentially expressed genes (DEGs). For confirmation, several DEGs were selected randomly to perform semi‐quantitative RT‐PCR analysis.

## Supporting information


**Fig. S1. *Xcc* strains grown in various media**
**(A)** Growth of *Xcc* strains in minimal medium NCM containing glucose, fructose, mannose, sorbose, rhamnose, ribose, xylose, arabinose, maltose, sucrose, citrate, malate and pyruvate respectively, as the sole carbon source. Overnight cultures of *Xcc* strains were collected, washed and resuspended in NCM liquid medium to an OD_600_ of 0.6. 2 μl of each strain was inoculated on the agar plates and incubated at 28 °C for 5 days.
**(B)** Growth curves of *Xcc* strains in nutrition rich medium NYG. Strains were inoculated into 100 ml NYG liquid medium, samples were taken in triplicate at intervals of 4 h, and plated on NYG agar. Bacterial CFU were counted after incubation at 28 °C for 3 days.
(C) Growth curves of *Xcc* strains in minimal medium MMX. Strains were inoculated into 100 ml MMX liquid medium, samples were taken in triplicate at intervals of 12 h.

**Fig. S2. HprK**_**Xcc**_ **is required for tolerance to SDS, NaCl, phenol and heavy metal cation in *Xcc***. Cultures of *Xcc* strains were diluted and plated on an NYG plate supplemented with different concentrations of NaCl (A) SDS (B) phenol (C) and heavy metal salt CdCl_2_ (D). Bacterial colonies were counted after incubation at 28 °C for 3 days. The representative results of only one out of three replicated experiments are presented.

**Fig. S3. Functional categories of DEGs in *hprK***_***Xcc***_ **/*ptsH* double mutant background**. Though 172 genes were found differentially expressed by two‐fold or more in *hprK*_*Xcc*_/*ptsH* double mutant, the expression of lots of DEGs in *hrpK*_*Xcc*_ mutant background was restored. Each bar represents the number of differential expressed genes in each category of *Xcc* 8004 genome. Grey bars indicate genes that were up‐regulated in mutant and white bars represent genes that were down‐regulated.

**Fig. S4. PtsH protein with Ser‐46 replacement has no activity**. *hprK*_*Xcc*_/*ptsH* double mutant ΔhprK_Xcc_ΔptsH were introduced with recombinant plasmids pLCptsH, pLCptsH_H15A_ and pLCptsH_S46A_ respectively. The resulted strains were tested for EPS production, activity of extracellular enzymes (protease and amylase) and cell motility (swimming) on the corresponding medium.

**Table S1.** Bacterial strains and plasmids used in this work.
**Table S2.** List of genes differentially expressed in ΔhprK_Xcc_ and ΔhprK_Xcc_/ptsH mutant backgrounds compared to wild‐type.
**Table S3.** Confirmation of the gene expression profile data of the hprK_Xcc_ mutant by semi‐quantitative RT‐PCR.
**Table S4.** Confirmation of the gene expression profile data of the hprK_Xcc_/ptsH double mutant by semi‐quantitative RT‐PCR.
**Table S5.** Primers used in this work.
