# Supplementary figures and images for: HprKXcc is a serine kinase that regulates virulence in the Gram‐negative phytopathogen Xanthomonas campestris
Source: Environ Microbiol. 2019 Jul 30;21(12):4504–20. doi: 10.1111/1462-2920.14740 (PMC6916182; doi:10.1111/1462-2920.14740)

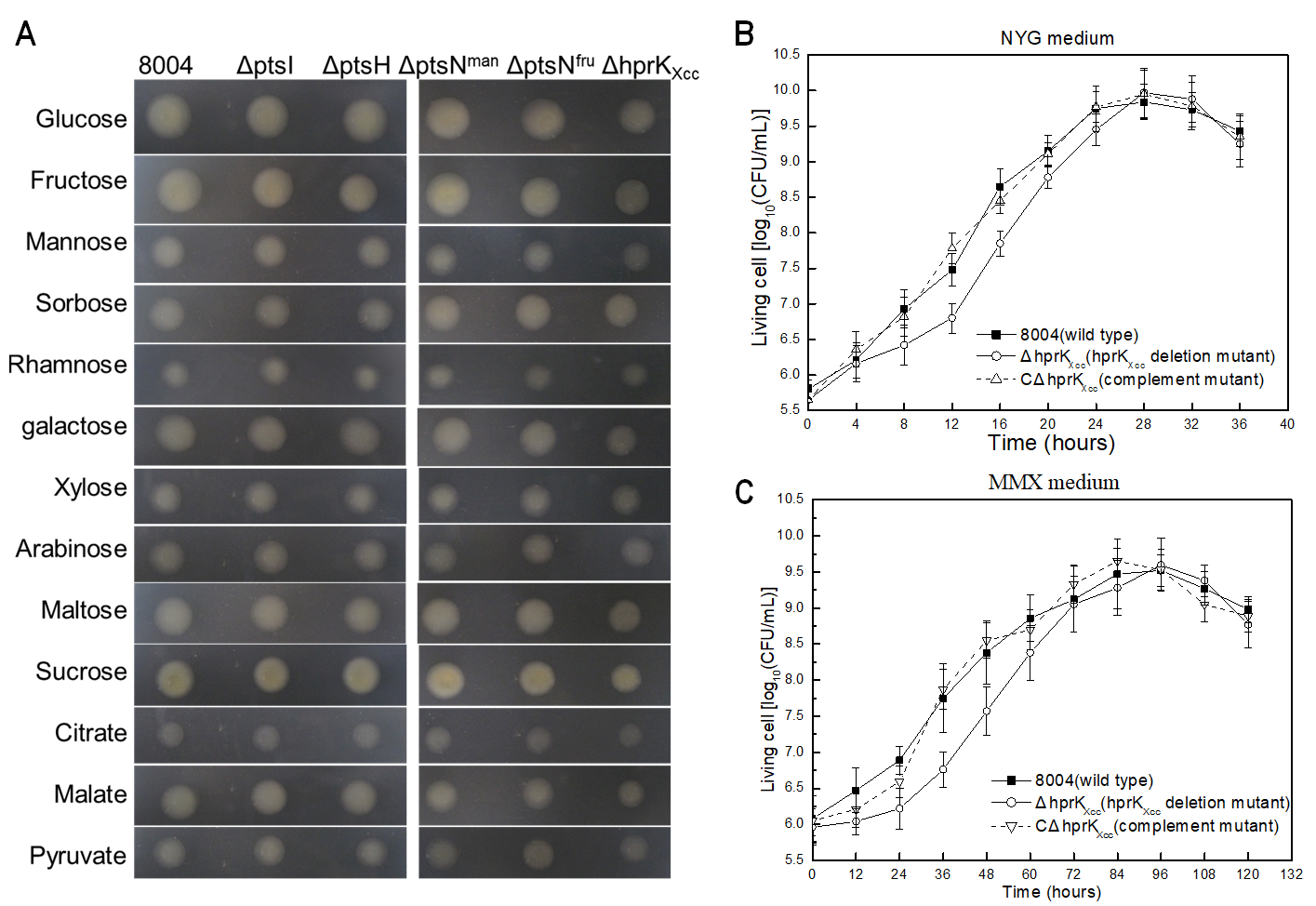

Supplement: Supplementary file 1 — Fig. S1. Xcc strains grown in various media (A) Growth of Xcc strains in minimal medium NCM containing glucose, fructose, mannose, sorbose, rhamnose, ribose, xylose, arabinose, maltose, sucrose, citrate, malate and pyruvate respectively, as the sole carbon source. Overnight cultures of Xcc strains were collected, washed and resuspended in NCM liquid medium to an OD600 of 0.6. 2 μl of each strain was inoculated on the agar plates and incubated at 28 °C for 5 days. (B) Growth curves of Xcc strains in nutrition rich medium NYG. Strains were inoculated into 100 ml NYG liquid medium, samples were taken in triplicate at intervals of 4 h, and plated on NYG agar. Bacterial CFU were counted after incubation at 28 °C for 3 days. (C) Growth curves of Xcc strains in minimal medium MMX. Strains were inoculated into 100 ml MMX liquid medium, samples were taken in triplicate at intervals of 12 h. [file EMI-21-4504-s001.tif]

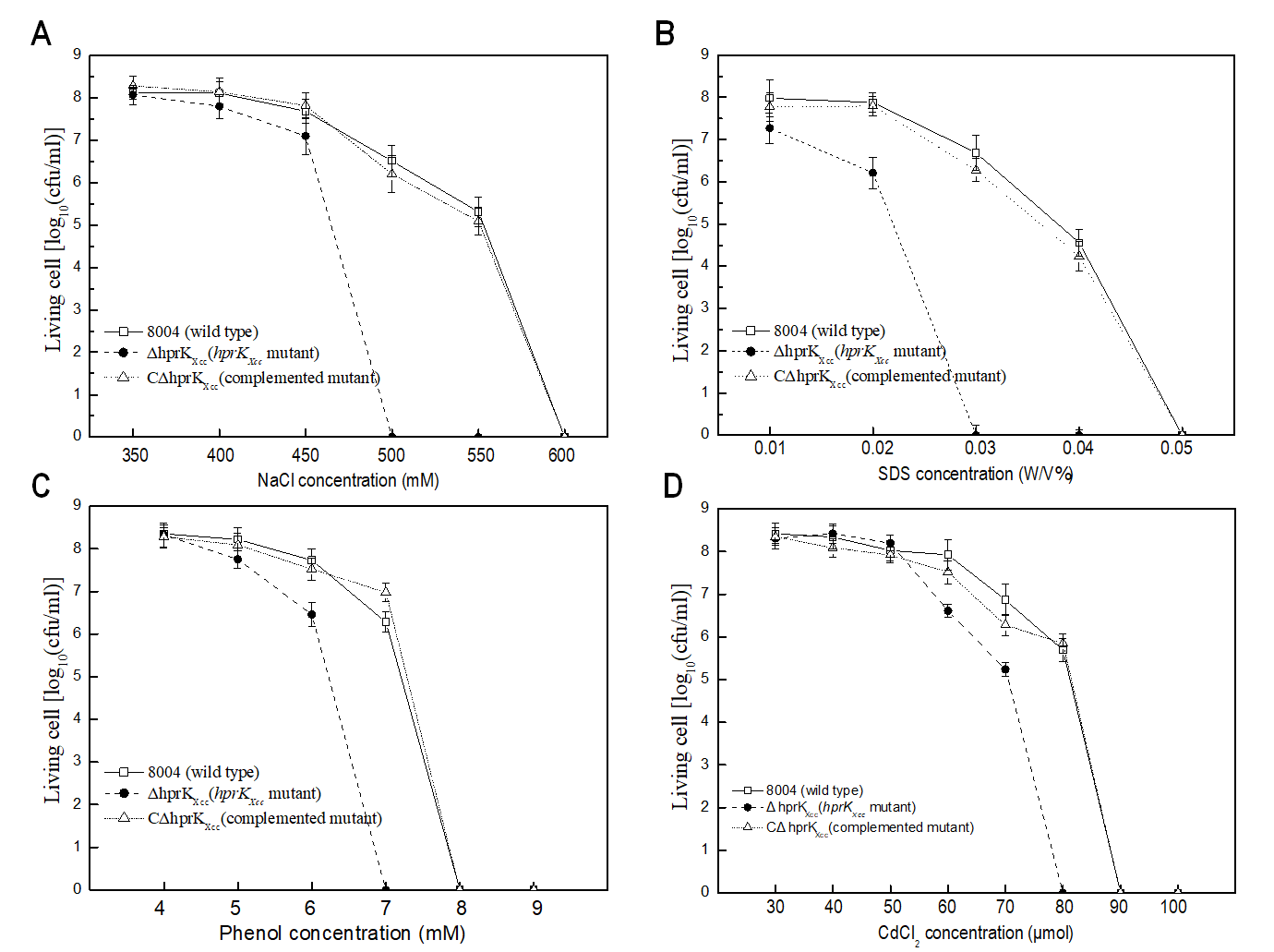

Supplement: Supplementary file 2 — Fig. S2. HprK Xcc is required for tolerance to SDS, NaCl, phenol and heavy metal cation in Xcc. Cultures of Xcc strains were diluted and plated on an NYG plate supplemented with different concentrations of NaCl (A) SDS (B) phenol (C) and heavy metal salt CdCl2 (D). Bacterial colonies were counted after incubation at 28 °C for 3 days. The representative results of only one out of three replicated experiments are presented. [file EMI-21-4504-s002.tif]

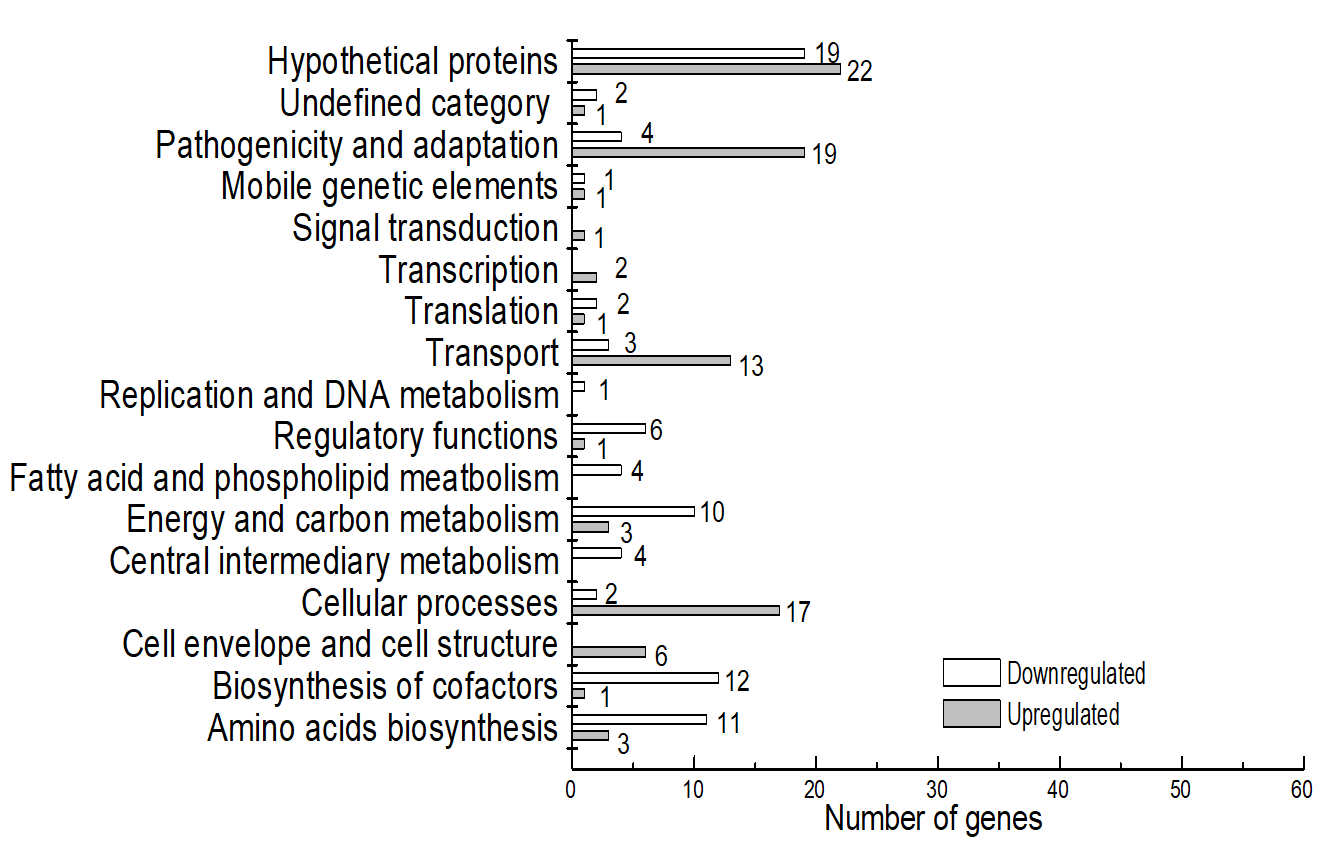

Supplement: Supplementary file 3 — Fig. S3. Functional categories of DEGs in hprK Xcc /ptsH double mutant background. Though 172 genes were found differentially expressed by two‐fold or more in hprK Xcc/ptsH double mutant, the expression of lots of DEGs in hrpK Xcc mutant background was restored. Each bar represents the number of differential expressed genes in each category of Xcc 8004 genome. Grey bars indicate genes that were up‐regulated in mutant and white bars represent genes that were down‐regulated. [file EMI-21-4504-s003.tif]

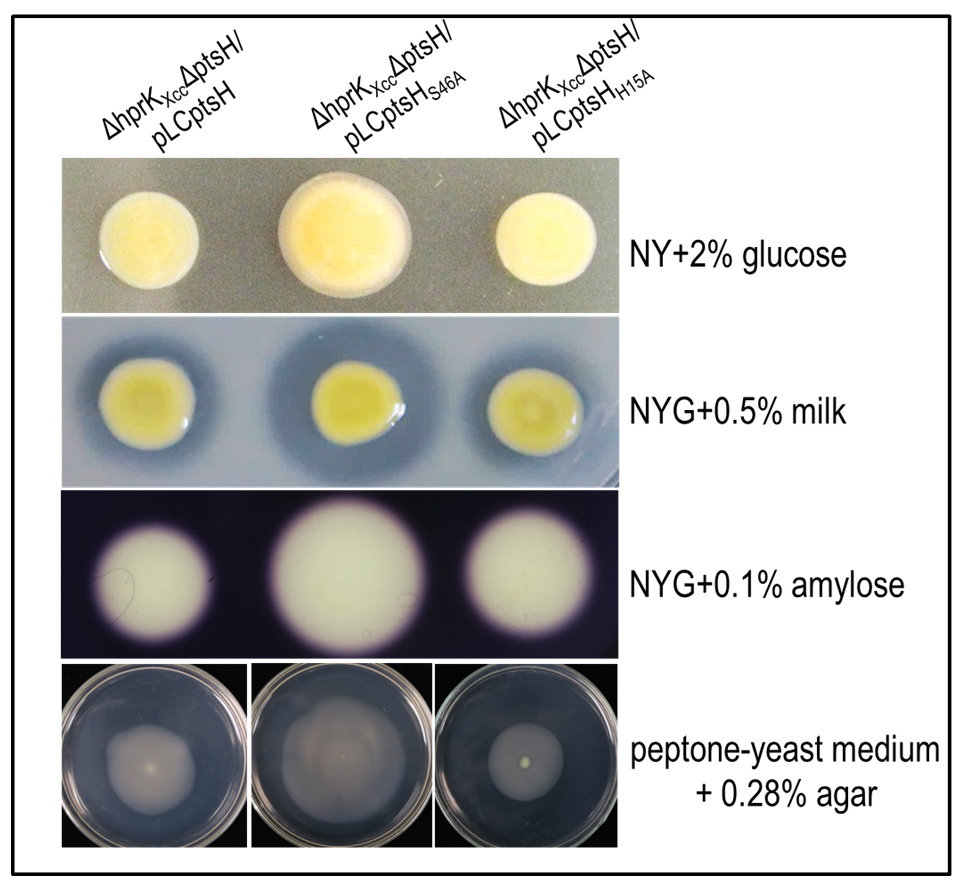

Supplement: Supplementary file 4 — Fig. S4. PtsH protein with Ser‐46 replacement has no activity. hprK Xcc/ptsH double mutant ΔhprKXccΔptsH were introduced with recombinant plasmids pLCptsH, pLCptsHH15A and pLCptsHS46A respectively. The resulted strains were tested for EPS production, activity of extracellular enzymes (protease and amylase) and cell motility (swimming) on the corresponding medium. [file EMI-21-4504-s004.tif]
