# Supplementary material for: HprKXcc is a serine kinase that regulates virulence in the Gram‐negative phytopathogen Xanthomonas campestris
Source: Environ Microbiol. 2019 Jul 30;21(12):4504–20. doi: 10.1111/1462-2920.14740 (PMC6916182; doi:10.1111/1462-2920.14740)
Supplement: Supplementary file 5 — Table S1. Bacterial strains and plasmids used in this work. Table S2. List of genes differentially expressed in ΔhprKXcc and ΔhprKXcc/ptsH mutant backgrounds compared to wild‐type. Table S3. Confirmation of the gene expression profile data of the hprKXcc mutant by semi‐quantitative RT‐PCR. Table S4. Confirmation of the gene expression profile data of the hprKXcc/ptsH double mutant by semi‐quantitative RT‐PCR. Table S5. Primers used in this work. [file EMI-21-4504-s005.doc]

**Table S1.** Strains and plasmids used in this study

| Strains or plasmids | Relevant characteristics | Reference or source |
| --- | --- | --- |
| *E. coli* |  |  |
| JM109 | *RecA*1*, endA*1*, gyrA*96*, thi, supE*44*, relA*1△ (*lac-proAB*)/F’ [*traD36, lacI*^q^, *lacZ* △M15] | Yanisch-Perron et al., 1985 |
| DH5α | Φ80△*lacZM*15 *recA1 endA1 deoR* | Gibco BRL, Life Technologies |
| M15 | *lac ara gal mtl recA1 uvr1* [pREP4 *lacI* Kan^r^] | Qiagen |
| BL21(DE3) | *hsdS gal* (λcI*ts857ind*1 *S*am7 *nin5 lac*UV5-T7 gene 1) | Novagen |
| *X. campestris* pv. *campestris* |  |  |
| 8004 | Wild type, Rif^r^ | Daniels *et al*., 1984 |
| ΔptsI | As 8004, but *ptsI* deleted, non-polar effect. Rif^r^ |  |
| ΔptsH | As 8004, but *ptsH* deleted, non-polar effect. Rif^r^ | This work |
| ΔptsN^Man^ | As 8004, but *ptsN^Man^* deleted, non-polar effect. Rif^r^ | This work |
| ΔptsN^Ntr^ | As 8004, but *ptsN^Ntr^* deleted, non-polar effect. Rif^r^ | This work |
| ΔhprK_Xcc_ | As 8004, but *hprK_Xcc_* deleted, non-polar effect. Rif^r^ | This work |
| CΔhprK_Xcc_ | ΔhprK_Xcc_ harboring the recombinant plasmid pLChprK_Xcc_, Rif^r^ Tet^r^ | This work |
| ΔhprK_Xcc_ΔptsI | As 8004, but both *hprK_Xcc_* and *ptsI* deleted, non-polar effect. Rif^r^ | This work |
| ΔhprK_Xcc_ΔptsH | As 8004, but both *hprK_Xcc_* and *ptsH* deleted, non-polar effect. Rif^r^ | This work |
| ΔhprK_Xcc_ΔptsN^Man^ | As 8004, but both *hprK_Xcc_* and *ptsN^Man^* deleted, non-polar effect. Rif^r^ | This work |
| ΔhprK_Xcc_ΔptsN^Ntr^ | As 8004, but both *hprK_Xcc_* and *ptsN^Ntr^* deleted, non-polar effect. Rif^r^ | This work |
| ΔhprK_Xcc_ΔptsH/pLCptsH | ΔhprK_Xcc_ΔptsH harboring the recombinant plasmid pLCptsH. Rif^r^ Tet^r^ | This work |
| ΔhprK_Xcc_ΔptsH/pLCptsH_H15A_ | ΔhprK_Xcc_ΔptsH harboring the recombinant plasmid pLCptsH_H15A_. Rif^r^ Tet^r^ | This work |
| ΔhprK_Xcc_ΔptsH/pLCptsH_S46A_ | ΔhprK_Xcc_ΔptsH harboring the recombinant plasmid pLCptsH_S46A_. Rif^r^ Tet^r^ | This work |
| ΔhprK_Xcc_ΔptsH/pLCptsH_15/46A_ | ΔhprK_Xcc_ΔptsH harboring the recombinant plasmid pLCptsH_15/46A_. Rif^r^ Tet^r^ | This work |
| ΔptsH/pHisptsH_lac_ | ΔptsH harboring the recombinant plasmid pHisptsH_lac_. Rif^r^ Tet^r^ | This work |
| ΔptsH/pHisptsH_H15A_ | ΔptsH harboring the recombinant plasmid pHisptsH_H15A_. Rif^r^ Tet^r^ | This work |
| ΔptsH/pHisptsH_S46A_ | ΔptsH harboring the recombinant plasmid pHisptsH_S46A_. Rif^r^ Tet^r^ | This work |
| ΔptsH/pHisptsH_15/46A_ | ΔptsH harboring the recombinant plasmid pHisptsH_15/46A_. Rif^r^ Tet^r^ | This work |
| ΔhprK_Xcc_ΔptsH/pHisptsH_lac_ | ΔhprK_Xcc_ΔptsH harboring the recombinant plasmid pLC1308, Rif^r^ Tet^r^ | This work |
| ΔhprK_Xcc_ΔptsH/pHisptsH_H15A_ | ΔhprK_Xcc_ΔptsH harboring the recombinant plasmid pHisptsH_H15A_. Rif^r^ Tet^r^ | This work |
| ΔhprK_Xcc_ΔptsH/pHisptsH_S46A_ | ΔhprK_Xcc_ΔptsH harboring the recombinant plasmid pHisptsH_S46A_. Rif^r^ Tet^r^ | This work |
| ΔhprK_Xcc_ΔptsH/pHisptsH_15/46A_ | ΔhprK_Xcc_ΔptsH harboring the recombinant plasmid pHisptsH_15/46A_. Rif^r^ Tet^r^ | This work |
| ΔhprK_Xcc_ΔhrpG/pHishrpG*_lac_* | *hprK_Xcc_* and *hrpG* double deletion mutant strain ΔhprK_Xcc_∆hrpG harbouring pHis*hrpG_lac_* , Rif^r^ Tet^r^ | This work |
| NK2699/pR3MipH6 | mip_Xcc_ gene mutant NK2699 harboring pR3MipH6, Rif^r^, Kan^r^ ,Tc^r^ | Meng *et al*., 2011 |
| 8004/ pLCptsH | 8004 strain harboring recombinant plasmid pLCptsH. Rif^r^ Tet^r^ | This work |
| 8004/pBptsH | 8004 strain harboring recombinant plasmid pBptsH. Rif^r^ Km^r^ | This work |
| 8004/pHisptsH_ara_ | 8004 strain harboring recombinant plasmid pHis*ptsH_ara_*. Rif^r^ Km^r^ | This work |
| 8004/pHisptsH_lac_ | 8004 strain harboring recombinant plasmid pHis*ptsH_lac_*. Rif^r^ Tet^r^ | This work |
| Plasmids |  |  |
| pQE-30 | Expression vector, allow the production of fusion proteins containing amino terminal 6xHis-tagged sequences. Ampr | Qiagen, Germany |
| pQE-HprK_Xcc_ | pQE-30 containing a 948-bp fragment of *hprK_Xcc_* gene coding region. | This work |
| pET-32a | Expression vector carrying the N-terminal Trx-His-S tag; Amp^r^ | Novagen |
| pET-PtsH | pET-32a containing a 270-bp fragment of wild-type *ptsH* gene coding region | This work |
| pET-PtsH_S46A_ | pET-32a containing a 270-bp DNA fragment of *Xcc* *ptsH* ORF in which the serine at position 46 was replaced by alanine. Tet^r^ | This work |
| pET-PtsH_S60A_ | pET-32a containing a 270-bp DNA fragment of *Xcc* *ptsH* ORF in which the serine at position 60 was replaced by alanine. Tet^r^ | This work |
| pET-PtsH_S46/60A_ | pET-32a containing a 270-bp DNA fragment of *Xcc* *ptsH* ORF in which the serines at position 46 and 60 were replaced by alanines. Tet^r^ | This work |
| pET-PtsH_S46/60T_ | pET-32a containing a 270-bp DNA fragment of *Xcc* *ptsH* ORF in which the serines at position 46 and 60 were replaced by threonines. Tet^r^ | This work |
| pLAFR3 | Broad host range cloning vector, Tet^r^ | Staskawicz *et al*., 1987 |
| pRK2073 | Helper plasmid, Tra+, Mob+, ColE1, Spc^r^. | Leong et al., 1982 |
| pK18*mobsacB* | pUC18 derivative, *lacZα*, *sacB*, Kan^r^, *mob* site. Allelic exchange vector (Suicidal vector carrying sacB gene for mutagenesis) | Schäfer *et al*., 1994 |
| pK18*mob* | pUC18 derivative, *lacZα* Kan^r^, *mob* site. Suicide plasmid in *Xcc*. | Schäfer *et al*., 1994 |
| pBBad22K | Km^r^, L-arabinose-inducible broad host range vector based on the pBBR1MCS-4 replicon | Sukchawalit et al., 1999 |
| pBptsH | pBBad22K containing 270-bp *ptsH* coding sequence. | This work |
| pKptsH | The suicide plasmid pK18mob containing 270-bp *ptsH* coding sequence. | This work |
| pLChprK_Xcc_ | 1201 bp DNA fragment containing the full *hprK_Xcc_* gene of *Xcc* strain cloned into the plasmid pLAFR6. Tet^r^ | This work |
| pLCptsH | 270 bp DNA fragment of *ptsH* (*XC_1305*) ORF of *Xcc* strain cloned into the plasmid pLAFR3. Tet^r^ | This work |
| pLCptsH_H15A_ | 270 bp DNA fragment of *Xcc* *ptsH* ORF in which the histidine at position 15 was replaced by alanine cloned into the plasmid pLAFR3. Tet^r^ | This work |
| pLCptsH_S46A_ | 270 bp DNA fragment of *Xcc* *ptsH* ORF in which the serine at position 46 was replaced by alanine cloned into the plasmid pLAFR3. Tet^r^ | This work |
| pLCptsH_15/46A_ | 270 bp DNA fragment of *Xcc* *ptsH* ORF in which both the histidine at position 15 and serine at position 46 were replaced by alanines cloned into the plasmid pLAFR3. Tet^r^ | This work |
| pHisptsH_lac_ | pLAFR3 containing HPr (PtsH) coding sequence with 6xHis tag in its C-terminal. Tet^r^ | This work |
| pHisptsH_ara_ | pBBad22K containing HPr (PtsH) coding sequence with 6xHis tag in its C-terminal. Km^r^ | This work |
| pHisptsH_H15A_ | pLAFR3 containing PtsH (HPr) coding sequence in which alanine substitute the histidine at position 15 fused with 6xHis tag in its C-terminal. Tet^r^ | This work |
| pHisptsH_S46A_ | pLAFR3 containing PtsH (HPr) coding sequence in which alanine substitute the serine at position 46 fused with 6xHis tag in its C-terminal. Tet^r^ | This work |
| pHisptsH_15/46A_ | pLAFR3 containing PtsH (HPr) coding sequence in which alanines substitute the histidine at position 15 and serine at position 46 fused with 6xHis tag in its C-terminal. Tet^r^ | This work |
| pR3MipH6 | pLAFR3 containing the *mip_Xcc_* coding sequence with 6xHis tag in its C-terminal, Tc^r^ | Meng et al. 2011 |

**References**

Daniels, M. J., Barber, C. E., Turner, P. C., Sawczyc, M. K., Byrde, R. J. & Fielding, A. H*.* Cloning of genes involved in pathogenicity of *Xanthomonas campestris* pv. *campestris* using the broad host range cosmid pLAFR1. *EMBO J.* **3**, 3323–3328 (1984).

Leong, S. A., Ditta, G. S. & Helinski, D. R. Heme biosynthesis in *Rhizobium*. Identification of a cloned gene coding for delta-aminolevulinic acid synthetase from *Rhizobium meliloti*. *J. Biol. Chem.* **257**, 8724–8730 (1982).

Schäfer, A., Tauch, A., Jäger, W., Kalinowski, J., Thierbach, G. & Pühler, A. Small mobilizable multi-purpose cloning vectors derived from the *Escherichia coli* plasmids pK18 and pK19: selection of defined deletions in the chromosome of *Corynebacterium glutamicum*. *Gene* **145**: 69–73 (1994).

Staskawicz, B., Dahlbeck, D., Keen, N. & Napoli, C. Molecular characterization of cloned avirulence genes fromrace 0 and race 1 of *Pseudomonas syringae* pv. *glycinea*. *J Bacteriol* **169**: 5789–5794 (1987).

Yanisch-Perron, C., Vieira, J. & Messing, J. Improved M13 phage cloning vectors and host strains: nucleotide sequences of the M13mp18 and pUC19 vectors. *Gene* **33**, 103–119 (1985).

**Table S2.** The up- and down-regulated differential expressed genes of the *hprK_Xcc_* mutant ∆hprK_Xcc_ and *hprK_Xcc_*/*ptsH* double mutant ∆hprK_Xcc_∆ptsH cultured in the NYG medium.

| Function Category | Gene ID | Name | Annotation | fold change  ∆hprK_Xcc_ | fold change  ∆hprK_Xcc_∆HPr*^-^* |
| --- | --- | --- | --- | --- | --- |
| Amino acids biosynthesis | *XC_1575* | *trpB* | tryptophan synthase beta chain | -2.25 | -2.16 |
|  | *XC_1577* | *trpA* | tryptophan synthase alpha chain | -2.38 | -2.13 |
|  | *XC_2175* | *nasE* | Nitrite assimilation small subunit | -2.27 | -9.25 |
|  | *XC_2374* | *hisI* | phosphoribosyl-AMP cyclohydrolase/phosphoribosyl-ATP pyrophosphatase bifunctional enzyme | -3.2 | -3.18 |
|  | *XC_2375* | *hisF* | bifunctionalaspartokinase/homoserine dehydrogenase I | -3.29 | -2.73 |
|  | *XC_2376* | *hisA* | phosphoribosylformimino-5-aminoimidazole carboxam | -3.76 | -2.75 |
|  | *XC_2377* | *hisH* | amidotransferase | -3.29 | -5.86 |
|  | *XC_2378* | *hisB* | imidazoleglycerolphosphate dehydratase/histidinol-phosphate phosphatase bifunctional enzyme | -3.20 | -2.71 |
|  | *XC_2379* | *hisC* | histidinol-phosphate aminotransferase | -3.43 | -2.99 |
|  | *XC_2380* | *hisD* | histidinol dehydrogenase | -4.50 | -2.41 |
|  | *XC_2381* | *hisG* | ATP phosphoribosyltransferase | -2.99 | -2.13 |
|  | *XC_1251* | *metA* | homoserine O-acetyltransferase | +8.69 | +5.86 |
|  | *XC_1252* | *metB* | cystathionine gamma-synthase | NO | +3.84 |
|  | *XC_2724* | *metH1* | 5-methyltetrahydrofolate-homocysteine methyl transferase | NO | +2.20 |
| Biosynthesis of cofactors, prosthetic groups, carriers | *XC_0400* | *bioB* | biotin synthase | NO | -2.27 |
|  | *XC_0575* | *mdcC* | malonate decarboxylase gamma subunit | -2.27 | -2.03 |
|  | *XC_0576* | *mdcE* | malonate decarboxylase | -4.17 | -2.76 |
|  | *XC_0577* | *citG* | CitG protein | -3.12 | -2.11 |
|  | *XC_0983* | *cysG* | siroheme synthase | -2.04 | -2.43 |
|  | *XC_1169* | *pqqE* | PqqE protein | -2.11 | -2.08 |
|  | *XC_1170* | *pqqC/D* | PqqC/D protein | -2.27 | -2.20 |
|  | *XC_1335* | *apbE* | thiamine biosynthesis lipoprotein ApbE precursor | -2.35 | NO |
|  | *XC_1545* | *oar* | Oar protein | NO | -2.04 |
|  | *XC_2468* | *panB* | 3-methyl-2-oxobutanoate hydroxymethyltransferase | NO | -2.14 |
|  | *XC_2555* |  | methyltransferase | -2.25 | -2.04 |
|  | *XC_3157* |  | hydroxylase molybdopterin-containing subunit | NO | -2.03 |
|  | *XC_3751* | *entB* | isochorismatase-like protein | -2.46 | NO |
|  | *XC_4045* | *hemB* | delta-aminolevulinic acid dehydratase | -2.83 | -2.39 |
|  | *XC_2857* | *pru* | protein U | NO | +2.23 |
| Cell envelope and cell structure | *XC_0609* |  | inner membrane protein | -2.11 | NO |
|  | *XC_0970* | *ompW* | outer membrane protein | -3.73 | -2.93 |
|  | *XC_1469* |  | inner membrane protein | -2.43 | NO |
|  | *XC_2151* | *mreD* | L-sorbosone dehydrogenase | -2.62 | NO |
|  | *XC_3884* | *yiaA* | membrane protein | -3.32 | -2.41 |
|  | *XC_0232* | *ddlB* | pre-pilin like leader sequence | +2.00 | NO |
|  | *XC_0939* | *pilO* | fimbrial assembly membrane protein | +2.50 | NO |
|  | *XC_0940* | *pilN* | fimbrial assembly membrane protein | +2.00 | NO |
|  | *XC_0941* | *pilM* | fimbrial assembly membrane protein | +2.48 | NO |
|  | *XC_1056* | *pilD* | type IV pre-pilin leader peptidase | +2.38 | +2.04 |
|  | *XC_1057* | *pilC* | fimbrial assembly protein | NO | +2.46 |
|  | *XC_1621* | *fimT* | pre-pilin like leader sequence | +3.40 | +3.18 |
|  | *XC_1622* | *pilV* | pre-pilin leader sequence | +4.62 | +4.08 |
|  | *XC_1624* | *pilX* | PilX protein | +2.55 | +2.43 |
|  | *XC_1626* | *pilE1* | type IV pilin | +2.30 | +2.43 |
| Cellular processes | *XC_0063* |  | regulatory protein cII | -2.19 | NO |
|  | *XC_0141* |  | alpha-amylase | -2.10 | NO |
|  | *XC_0142* |  | trehalose synthase | -2.08 | NO |
|  | *XC_0143* | *glgB1* | 1,4-alpha-glucan branching enzyme | -2.20 | NO |
|  | *XC_0424* |  | 4-alpha-glucanotransferase | -2.11 | NO |
|  | *XC_0970* | *ompW* | outer membrane protein | NO | -2.93 |
|  | *XC_1290* | *cheB* | protein-glutamate methylesterase | -2.83 | NO |
|  | *XC_1368* | *osmC* | osmotically inducible protein | -2.60 | NO |
|  | *XC_1412* | *cheW* | chemotaxis protein | -2.99 | NO |
|  | *XC_1413* | *mcp* | chemotaxis protein | -2.81 | NO |
|  | *XC_2245* | *fliC* | flagellar protein | -2.60 | +2.41 |
|  | *XC_2284* | *cheA* | chemotaxis related protein | -2.48 | NO |
|  | *XC_2302* | *cheY* | chemotaxis response regulator | -2.11 | +3.41 |
|  | *XC_2306* | *tsr* | chemotaxis protein | -2.11 | +2.02 |
|  | *XC_2311* | *tsr* | chemotaxis protein | -2.03 | +2.04 |
|  | *XC_2315* |  | methyl-accepting chemotaxis protein | -2.62 | NO |
|  | *XC_2318* | *cheW* | chemotaxis protein | -2.20 | +2.07 |
|  | *XC_2320* | *tsr* | chemotaxis protein | -2.16 | NO |
|  | *XC_2322* | *cheD* | chemotaxis protein | -2.01 | NO |
|  | *XC_2323* | *cheB* | glutamate methylesterase | -2.04 | NO |
|  | *XC_0609* |  | inner membrane protein | NO | +2.25 |
|  | *XC_2237* | *flgI* | flagellar protein | NO | +2.91 |
|  | *XC_2237* | *flgI* | flagellar protein | NO | +2.91 |
|  | *XC_2238* | *flgF* | flagellar protein | NO | +2.81 |
|  | *XC_2241* | *flgI* | flagellar protein | NO | +2.91 |
|  | *XC_2242* | *flgJ* | flagellar protein | NO | +2.19 |
|  | *XC_2243* | *flgK* | flagellar protein | NO | +2.11 |
|  | *XC_2280* | *fleN* | flagellar biosynthesis switch protein | NO | +2.83 |
|  | *XC_2282* | *cheY* | chemotaxis protein | NO | +3.86 |
|  | *XC_2283* | *cheZ* | chemotaxis related protein | NO | +2.03 |
| Central intermediary metabolism | *XC_0150* |  | L-fucose dehydrogenase | -2.69 | NO |
|  | *XC_0374* | *pobB* | phenoxybenzoate dioxygenase beta subunit | -3.10 | -2.17 |
|  | *XC_0375* | *vanA* | vanillate O-demethylase oxygenase subunit | -2.20 | -2.16 |
|  | *XC_0990* | *cysH* | 3'-phosphoadenosine 5'-phosphosulfate reductase | -3.14 | -3.92 |
|  | *XC_0991* | *cysI* | NADPH-sulfite reductase iron-sulfur protein | -2.64 | -2.75 |
|  | *XC_0993* | *cysD* | ATP sulfurylase small subunit | -2.31 | NO |
|  | *XC_2697* |  | thiosulfate sulfurtransferase | -2.04 | NO |
| Energy and carbon metabolism | *XC_0374* | *pobB* | phenoxybenzoate dioxygenase beta subunit | -3.10 | NO |
|  | *XC_0375* | *vanA* | vanillate O-demethylase oxygenase subunit | -2.20 | NO |
|  | *XC_1300* | *qxtB* | quinol oxidase, subunit II | -2.73 | -2.73 |
|  | *XC_1301* |  | quinol oxidase, subunit I | -2.62 | -2.62 |
|  | *XC_1384* |  | alcohol dehydrogenase | -2.00 | NO |
|  | *XC_1386* | *yagS* | oxidoreductase | -2.03 | -2.58 |
|  | *XC_1387* | *yagR* | oxidoreductase | -2.06 | -2.41 |
|  | *XC_1589* | *nuoA* | NADH-ubiquinone oxidoreductase NQO7 subunit | -2.07 | NO |
|  | *XC_1743* | *fruK* | 1-phosphofructokinase | -2.91 | -2.89 |
|  | *XC_2326* | *acnB* | aconitate hydratase 2 | NO | -2.04 |
|  | *XC_2573* | *dsbE* | C-type cytochrome biogenesis protein/thioredoxin | -2.68 | -2.31 |
|  | *XC_2659* | *gcd* | glucose dehydrogenase | -3.66 | -2.73 |
|  | *XC_2822* | *fumB* | fumarate hydratase | NO | -3.23 |
|  | *XC_3167* |  | oxidoreductase | -3.01 | NO |
|  | *XC_3170* |  | oxidoreductase | -2.55 | +2.19 |
|  | *XC_3740* |  | oxidoreductase | -3.01 | NO |
|  | *XC_3774* |  | Zn-dependent alcohol dehydrogenase | -2.85 | NO |
|  | *XC_4082* | *zwf* | glucose-6-phosphate 1-dehydrogenase | -2.07 | NO |
|  | *XC_1446* | *mocA* | oxidoreductase | 2.64 | NO |
|  | *XC_2800* | *dauE* | aklaviketone re | +6.19 | +2.63 |
| Fatty acid and phospholipidmeatbolism | *XC_0578* | *mdcH* | malonyl CoA-ACP transacylase | -2.27 | NO |
|  | *XC_1394* |  | phospholipase A1 | -2.87 | -2.87 |
|  | *XC_1395* | *plaS* | accessory protein | -2.83 | -2.83 |
|  | *XC_1408* | *cls* | cardiolipin synthase | -2.06 | -2.06 |
| Regulatory functions | *XC_0072* | *hilA* | transcriptional regulator | -2.48 | -2.39 |
|  | *XC_1280* |  | transcriptional regulator | NO | -2.30 |
|  | *XC_1745* |  | transcriptional regulator | NO | -2.64 |
|  | *XC_1909* |  | transcriptional regulator | NO | -2.85 |
|  | *XC_2157* |  | transcriptional regulator | -2.38 | -2.64 |
|  | *XC_3799* |  | transcriptional regulator | -2.16 | -6.06 |
|  | *XC_2723* |  | transcriptional regulator | +3.34 | +2.45 |
|  | *XC_4061* |  | transcriptional regulator | +2.17 | NO |
| Replication and DNA metabolism | *XC_0109* |  | ATP-dependent DNA ligase | -2.01 | NO |
|  | *XC_1808* | *lig3* | ATP-dependent DNA ligase | -2.08 | NO |
|  | *XC_2168* | *xthA2* | exodeoxyribonuclease III | -2.04 | -2.10 |
| Transport | *XC_1087* |  | ABC transporter ATP-binding subunit | -2.16 | NO |
|  | *XC_1152* |  | chloride channel | -2.43 | NO |
|  | *XC_1744* | *fruB* | multiphosphoryl transfer protein | NO | -2.39 |
|  | *XC_1887* |  | ABC transporter ATP-binding protein | -2.14 | -2.08 |
|  | *XC_2547* |  | ABC transporter ATP-binding protein | -2.28 | NO |
|  | *XC_2844* | *brf* | bacterioferritin | -2.14 | -2.99 |
|  | *XC_3201* |  | bacterioferritin | NO | -4.32 |
|  | *XC_3463* | *phuR* | outer membrane hemin receptor | -3.43 | +2.70 |
|  | *XC_3560* | *pnuC* | PnuC protein | -2.64 | NO |
|  | *XC_0642* | *fpvA* | ferripyoverdine receptor | +2.25 | +2.51 |
|  | *XC_0820* | *dctA* | C4-dicarboxylate transport protein | NO | +2.6 |
|  | *XC_0924* | *fhuE* | outer membrane receptor for ferric iron uptake | NO | +2.23 |
|  | *XC_0925* |  | outer membrane receptor for ferric iron uptake | NO | +3.23 |
|  | *XC_1241* | *btuB* | TonB-dependent receptor | +3.14 | +4.23 |
|  | *XC_1341* | *fhuA* | TonB-dependent receptor | NO | +2.45 |
|  | *XC_2295* |  | putative high-affinity Fe^2+^/Pb^2+^ permease | +3.05 | +3.12 |
|  | *XC_2708* | *pstS* | ABC transporter phosphate binding protein | +9.71 | +4.89 |
|  | *XC_2709* | *pstC* | ABC transporter phosphate permease | +5.47 | NO |
|  | *XC_2710* | *pstA* | ABC transporter phosphate permease | +3.92 | NO |
|  | *XC_2846* | *fhuA* | iron receptor | NO | +2.53 |
|  | *XC_3463* | *phuR* | outer membrane hemin receptor | NO | +2.71 |
|  | *XC_4044* | *piuB* | iron-uptake factor | NO | +2.25 |
| Translation | *XC_0294* | *amaB* | N-carbamyl-L-amino acid amidohydrolase | -2.13 | NO |
|  | *XC_0804* | *nonF* | NonF-related protein | -2.03 | NO |
|  | *XC_0945* | *rpmE* | 50S ribosomal protein L31 | -2.85 | -2.31 |
|  | *XC_0957* | *rpoZ* | RNA polymerase omega subunit | -2.36 | NO |
|  | *XC_1350* | *pfpI* | protease | -2.27 | NO |
|  | *XC_1422* |  | cysteine protease | -2.03 | NO |
|  | *XC_2568* |  | serine protease | -2.87 | NO |
|  | *XC_2972* | *mucD* | periplasmic protease | -2.45 | NO |
|  | *XC_3090* | *rpsT* | 30S ribosomal protein S20 | -2.23 | -2.04 |
|  | *XC_3329* | *rplX* | 50S ribosomal protein L24 | -2.33 | NO |
|  | *XC_3335* | *rplV* | 50S ribosomal protein L22 | -2.79 | NO |
|  | *XC_3337* | *rplB* | 50S ribosomal protein L2 | -2.55 | NO |
|  | *XC_3339* | *rplD* | 50S ribosomal protein L4 | -2.69 | NO |
|  | *XC_3340* | *rplC* | 50S ribosomal protein L3 | -2.58 | NO |
|  | *XC_3350* |  | serine protease | -2.13 | NO |
|  | *XC_0042* | *nonF* | NonF-related protein | +2.25 | +2.14 |
| Transcription | *XC_1904* |  | pseudouridylate synthase | -2.16 | NO |
|  | *XC_2281* | *fliA* | RNA polymerase sigma factor | -2.21 | NO |
|  | *XC_3643* | *rhlE* | ATP-dependent RNA helicase | -2.13 | NO |
|  | *XC_3850* | *rho* | transcription termination factor Rho | -2.19 | NO |
|  | *XC_0478* |  | anticodon nuclease | 2.10 | NO |
|  | *XC_0556* | *fecI* | RNA polymerase sigma factor | +2.58 | +3.94 |
|  | *XC_2251* | *rpoN* | RNA polymerase sigma-54 factor | +2.50 | +2.22 |
| Signal transduction | *XC_1686* |  | histidine kinase/response regulator hybrid protein | -2.00 | NO |
|  | *XC_0557* | *fecR* | transmembrane sensor | +2.20 | +2.64 |
|  | *XC_3272* | *phoB* | two-component system regulatory protein | +2.57 | NO |
| Mobile genetic elements | *XC_2111* | *gVIII* | major coat protein | -3.29 | NO |
|  | *XC_2121* | *gVII* | minor coat protein | -3.29 | NO |
|  | *XC_2115* | *orf112* | phage-related protein | +3.37 | +4.23 |
|  | *XC_2597* | *ISxac3* | ISxac3 transposase | NO | -2.50 |
|  | *XC_4280* | *IS1481* | IS1481 transposase | +2.62 | NO |
| Pathogenicity and adaptation | *XC_0028* | *egl* | cellulase | -2.45 | NO |
|  | *XC_0153* | *xynB* | xylanase | -2.45 | NO |
|  | *XC_0625* |  | cellulase | -2.81 | NO |
|  | *XC_0738* | *xcsC* | type II secretion system protein C | -2.38 | NO |
|  | *XC_0745* | *xcsJ* | type II secretion system protein J | -2.53 | NO |
|  | *XC_0748* | *xcsM* | type II secretion system protein M | -2.41 | NO |
|  | *XC_0851* | *virP* | VirP protein | -1.99 | NO |
|  | *XC_1005* |  | 1,4-beta-cellobiosidase | -2.01 | NO |
|  | *XC_1085* |  | glutathione transferase | -2.23 | NO |
|  | *XC_1298* | *pelB* | pectate lyase II | -2.61 | NO |
|  | *XC_1411* | *vieA* | response regulator | -2.24 | NO |
|  | *XC_1447* |  | extracellular serine protease | -2.11 | NO |
|  | *XC_1450* |  | extracellular serine protease | -6.67 | NO |
|  | *XC_1658* | *gumB* | GumB protein | -2.43 | NO |
|  | *XC_1659* | *gumC* | GumC protein | -2.05 | NO |
|  | *XC_1660* | *gumD* | GumD protein | -2.07 | NO |
|  | *XC_1661* | *gumE* | gumE protein | -2.08 | NO |
|  | *XC_1662* | *gumF* | GumF protein | -2.15 | NO |
|  | *XC_1811* | *acvB* | virulence protein | -2.10 | NO |
|  | *XC_1946* | *cpo* | non-heme chloroperoxidase | -2.39 | NO |
|  | *XC_2324* | *pdeA* | c-di-GMP phosphodiesterase A | -2.93 | NO |
|  | *XC_2329* | *rpfA* | aconitase | -2.38 | -2.87 |
|  | *XC_2699* |  | peptidyl-prolyl cis-trans isomerase | -2.46 | NO |
|  | *XC_2834* | *bglS* | beta-glucosidase | -3.84 | -2.55 |
|  | *XC_3008* | *hrpB4* | HrpB4 protein | -2.23 | +2.01 |
|  | *XC_3153* | *tcmJ* | tetracenomycin polyketide synthesis protein | -2.07 | NO |
|  | *XC_3376* |  | extracellular protease | -2.20 | NO |
|  | *XC_3377* |  | extracellular protease | -2.04 | NO |
|  | *XC_3591* | *pel* | pectate lyase | -2.17 | -2.04 |
|  | *XC_3657* | *copB* | copper resistance protein B precursor | -2.11 | NO |
|  | *XC_3754* |  | Mn-containing catalase | -4.69 | -4.56 |
|  | *XC_4064* | *czcD* | heavy metal transporter | -2.31 | NO |
|  | *XC_4256* | *fusE* | fusaric acid resistance protein | -3.18 | NO |
|  | *XC_4256* | *fusE* | fusaric acid resistance protein | -2.58 | NO |
|  | *XC_0705* | *peh-1* | endopolygalacturonase | NO | +2.13 |
|  | *XC_0784* |  | cellulase S | +2.25 | +3.71 |
|  | *XC_1057* | *pilC* | fimbrial assembly protein | +2.51 | +2.46 |
|  | *XC_1119* | *bla* | beta lactamase | NO | +3.53 |
|  | *XC_1120* | *xynB* | xylanase | NO | +2.25 |
|  | *XC_1398* | *cstA* | carbon starvation protein A | +2.10 | NO |
|  | *XC_1625* |  | PilY1 protein | +2.76 | +2.62 |
|  | *XC_2082* | *avrBs1.1* | avirulence protein | +2.83 | +2.22 |
|  | *XC_2160* | *yapH* | YapH protein | +2.01 | +3.32 |
|  | *XC_3001* | *hpa2* | Hpa2 protein | +2.14 | +2.33 |
|  | *XC_3002* | *hpa1* | Hpa1 protein | +3.78 | +6.23 |
|  | *XC_3003* | *hrcC* | HrcC protein | NO | +2.11 |
|  | *XC_3009* | *hrcJ* | HrcJ protein | +2.22 | +2.20 |
|  | *XC_3011* | *hrpB1* | HrpB1 protein | +2.11 | +5.28 |
|  | *XC_3012* | *hrcU* | HrcU protein | +5.24 | +3.05 |
|  | *XC_3014* | *hpaP* | HpaP protein | NO | +3.71 |
|  | *XC_3018* | *hpaA* | HpaA protein | +2.17 | +2.11 |
|  | *XC_3019* | *hrpD5* | HrpD5 protein | +2.45 | +2.83 |
|  | *XC_3021* | *hrpE* | HrpE protein | +2.64 | +2.01 |
| Undefined category | *XC_1470* | *visC* | putative monooxygenase NMA2164 | -3.46 | -2.51 |
|  | *XC_3972* |  | hydrolase or peptidase | -2.38 | NO |
|  | *XC_1699* | *soxA* | sarcosine oxidase alpha subunit | +3.51 | +3.61 |
|  | *XC_3158* |  | hydrolase or peptidase | +2.07 | -2.39 |
| conserved hypothetical protein | *XC_0025* |  | conserved hypothetical protein | -2.91 | NO |
|  | *XC_0069* |  | conserved hypothetical protein | -2.23 | -3.2 |
|  | *XC_0076* |  | conserved hypothetical protein | -2.11 | -2.51 |
|  | *XC_0624* |  | conserved hypothetical protein | -4.56 | NO |
|  | *XC_0723* |  | hypothetical protein | -2.28 | NO |
|  | *XC_1289* |  | conserved hypothetical protein | -5.70 | -3.39 |
|  | *XC_1336* |  | conserved hypothetical protein | -2.17 | +3.12 |
|  | *XC_1348* |  | conserved hypothetical protein | +3.25 | +2.51 |
|  | *XC_1388* |  | conserved hypothetical protein | -2.22 | -2.77 |
|  | *XC_1576* |  | conserved hypothetical protein | -2.14 | NO |
|  | *XC_1687* |  | conserved hypothetical protein | -6.97 | -3.32 |
|  | *XC_1807* |  | conserved hypothetical protein | -2.64 | NO |
|  | *XC_1826* |  | conserved hypothetical protein | -2.68 | -2.16 |
|  | *XC_1967* |  | conserved hypothetical protein | -2.36 | NO |
|  | *XC_2037* |  | conserved hypothetical protein | NO | -2.95 |
|  | *XC_2164* |  | conserved hypothetical protein | -2.38 | NO |
|  | *XC_2182* |  | conserved hypothetical protein | -3.71 | NO |
|  | *XC_2189* |  | conserved hypothetical protein | -2.39 | NO |
|  | *XC_2230* |  | conserved hypothetical protein | -3.23 | NO |
|  | *XC_2301* |  | conserved hypothetical protein | -2.01 | NO |
|  | *XC_2312* |  | conserved hypothetical protein | -2.06 | NO |
|  | *XC_2319* |  | conserved hypothetical protein | -2.39 | NO |
|  | *XC_2411* |  | conserved hypothetical protein | -2.87 | NO |
|  | *XC_2412* |  | conserved hypothetical protein | -2.66 | NO |
|  | *XC_2414* |  | conserved hypothetical protein | -4.59 | NO |
|  | *XC_2415* |  | conserved hypothetical protein | -16.0 | NO |
|  | *XC_2581* |  | conserved hypothetical protein | -5.10 | -2.31 |
|  | *XC_2721* |  | hypothetical protein | -2.58 | NO |
|  | *XC_2821* |  | conserved hypothetical protein | NO | -2.36 |
|  | *XC_2921* |  | conserved hypothetical protein | -3.73 | NO |
|  | *XC_2931* |  | conserved hypothetical protein | -2.31 | -2.16 |
|  | *XC_3171* |  | conserved hypothetical protein | -3.29 | NO |
|  | *XC_3422* |  | conserved hypothetical protein | NO | -2.19 |
|  | *XC_3462* |  | conserved hypothetical protein | -4.53 | 2.57 |
|  | *XC_3752* |  | conserved hypothetical protein | -3.16 | NO |
|  | *XC_3753* |  | conserved hypothetical protein | -3.14 | NO |
|  | *XC_3755* |  | conserved hypothetical protein | -3.87 | -3.56 |
|  | *XC_3770* |  | conserved hypothetical protein | -2.04 | NO |
|  | *XC_3772* |  | conserved hypothetical protein | -3.92 | -2.00 |
|  | *XC_3773* |  | conserved hypothetical protein | -5.58 | -2.89 |
|  | *XC_3775* |  | conserved hypothetical protein | -2.17 | NO |
|  | *XC_3784* |  | conserved hypothetical protein | NO | -2.60 |
|  | *XC_3855* |  | conserved hypothetical protein | -3.41 | NO |
|  | *XC_3856* |  | conserved hypothetical protein | -2.27 | NO |
|  | *XC_3862* |  | conserved hypothetical protein | -2.04 | NO |
|  | *XC_3870* |  | conserved hypothetical protein | -3.58 | -2.95 |
|  | *XC_3883* |  | conserved hypothetical protein | -5.39 | -2.91 |
|  | *XC_3971* |  | conserved hypothetical protein | -3.92 | NO |
|  | *XC_3974* |  | conserved hypothetical protein | -5.50 | -2.17 |
|  | *XC_3989* |  | conserved hypothetical protein | -2.18 | NO |
|  | *XC_4012* |  | conserved hypothetical protein | -5.03 | NO |
|  | *XC_4245* |  | conserved hypothetical protein | -2.28 | NO |
|  | *XC_4260* |  | conserved hypothetical protein | -2.51 | NO |
|  | *XC_4265* |  | conserved hypothetical protein | -2.50 | NO |
|  | *XC_0111* |  | conserved hypothetical protein | +3.32 | NO |
|  | *XC_0130* |  | conserved hypothetical protein | +2.71 | NO |
|  | *XC_0817* |  | conserved hypothetical protein | NO | +2.31 |
|  | *XC_1147* |  | conserved hypothetical protein | +2.57 | +5.58 |
|  | *XC_1242* |  | conserved hypothetical protein | +2.06 | NO |
|  | *XC_1382* |  | conserved hypothetical protein | +3.18 | NO |
|  | *XC_1424* |  | conserved hypothetical protein | +2.77 | +2.97 |
|  | *XC_1585* |  | conserved hypothetical protein | +2.19 | NO |
|  | *XC_1623* |  | conserved hypothetical protein | +3.39 | +7.78 |
|  | *XC_2138* |  | conserved hypothetical protein | +3.07 | +3.41 |
|  | *XC_2401* |  | conserved hypothetical protein | +2.36 | NO |
|  | *XC_2416* |  | conserved hypothetical protein | +12.73 | +13.45 |
|  | *XC_2422* |  | conserved hypothetical protein | +2.28 | +2.11 |
|  | *XC_2424* |  | conserved hypothetical protein | +2.25 | +3.29 |
|  | *XC_2570* |  | conserved hypothetical protein | +2.99 | +7.78 |
|  | *XC_2631* |  | conserved hypothetical protein | NO | +2.35 |
|  | *XC_2632* |  | conserved hypothetical protein | NO | +3.34 |
|  | *XC_2633* |  | conserved hypothetical protein | NO | +3.63 |
|  | *XC_3177* |  | conserved hypothetical protein | +2.08 | NO |
|  | *XC_3423* |  | conserved hypothetical protein | +3.63 | NO |
|  | *XC_3461* |  | conserved hypothetical protein | NO | +3.39 |
|  | *XC_3465* |  | conserved hypothetical protein | +2.19 | +3.46 |
|  | *XC_4034* |  | conserved hypothetical protein | NO | +2.53 |
|  | *XC_4035* |  | conserved hypothetical protein | +2.20 | +2.85 |
|  | *XC_4153* |  | conserved hypothetical protein | NO | +2.88 |
|  | *XC_4199* |  | hypothetical protein | +2.85 | +2.57 |

Note: False discovery rate (FDR) =0.05 and absolute value of log2FC(log2foldchange) =1 (equivalent to a fold change of 2) were used as the cut off values.“+” represents gene up-regulated in the ∆hprK_Xcc_ and ∆hprK_Xcc_∆ptsH mutants, and “-”represents gene down-regulated. Overlapping DEGs in both ∆hprK_Xcc_ and ∆hprK_Xcc_∆ptsH mutants are highlighted with light gray background, DEGs in ∆hprK_Xcc_ mutant but not in ∆hprK_Xcc_∆ptsH mutant are highlighted with yellow background.

**Table S3. Confirmation of the gene expression profile data of the *hprK_Xcc_* mutant by semi-quantitative RT-PCR.**

| ID | Gene | Description | Expresstion level in *hprK_Xcc_* mutant background | Semi RT-PCR  △hprK_Xcc_ /wt |
| --- | --- | --- | --- | --- |
| *XC_1575* | *trpB* | tryptophan synthase beta chain | -2.25↓ | 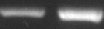 |
| *XC_2375* | *hisF* | bifunctionalaspartokinase | -3.29↓ | 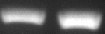 |
| *XC_0970* | *ompW* | outer membrane protein | -3.73↓ | 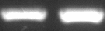 |
| *XC_1621* | *fimT* | pre-pilin like leader sequence | +3.40↑ | 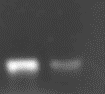 |
| *XC_2302* | *cheY* | chemotaxis response regulator | -2.11↓ | 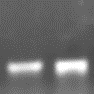 |
| *XC_0993* | *cysD* | ATP sulfurylase small subunit | -2.31↓ | 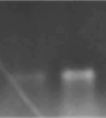 |
| *XC_3740* |  | oxidoreductase | -3.01↓ | 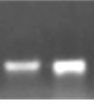 |
| *XC_2723* |  | transcriptional regulator | +3.34↑ | 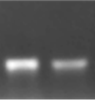 |
| *XC_1887* |  | ABC transporter ATP-binding protein | -2.14↓ | 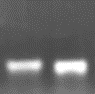 |
| *XC_3560* | *pnuC* | PnuC protein | -2.64↓ | 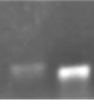 |
| *XC_2709* | *pstC* | ABC transporter phosphate permease | +5.47↑ | 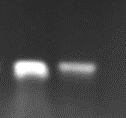 |
| *XC_1350* | *pfpI* | protease | -2.27↓ | 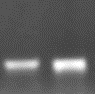 |
| *XC_0556* | *fecI* | RNA polymerase sigma factor | +2.58↑ | 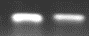 |
| *XC_0557* | *fecR* | transmembrane sensor | +2.20↑ | 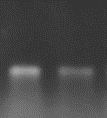 |
| *XC_0028* | *egl* | cellulase | -2.45↓ | 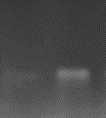 |
| *XC_0738* | *xcsC* | type II secretion system protein C | -2.38↓ | 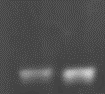 |
| *XC_1658* | *gumB* | GumB protein | -2.43↓ | 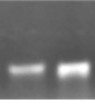 |
| *XC_2699* |  | peptidyl-prolyl cis-trans isomerase | -2.46↓ | 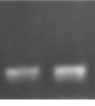 |
| *XC_3376* |  | extracellular protease | -2.20↓ | 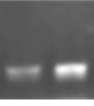 |
| *XC_3754* |  | Mn-containing catalase | -4.69↓ | 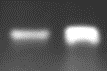 |
| 16S |  |  |  | 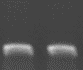 |

**Note: Validation of differentially expressed genes by semi quantitative reverse-transcription PCR (semi RT-PCR).** In this study, false discovery rate (FDR) =0.05 and absolute value of log_2_foldchange =1 (equivalent to a fold change of 2) were used as the cut off values. ↑: up-regulated; ↓: down-regulated.

**Table S4. Confirmation of the gene expression profile data of the *hprK_Xcc_*/*ptsH* double mutant by semi-quantitative RT-PCR.**

**Note: Validation of differentially expressed genes by semi quantitative reverse-transcription PCR (semi RT-PCR).** In this study, false discovery rate (FDR) =0.05 and absolute value of log_2_foldchange =1 (equivalent to a fold change of 2) were used as the cut off values. ↑: up-regulated; ↓: down-regulated.

| ID | Gene | Description | Expresstion level in *hprK_Xcc_*/*ptsH* double mutant background | Semi RT-PCR  △hprK_Xcc_△ptsH/wt |
| --- | --- | --- | --- | --- |
| *XC_1575* | *trpB* | tryptophan synthase beta chain | -2.16↓ | 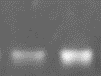 |
| *XC_2375* | *hisF* | bifunctionalaspartokinase | -2.73↓ | 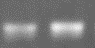 |
| *XC_0970* | *ompW* | outer membrane protein | -2.93↓ | 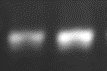 |
| *XC_2857* | *pru* | protein U | +2.23↑ | 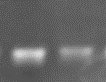 |
| *XC_1621* | *fimT* | pre-pilin like leader sequence | +3.18↑ | 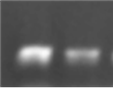 |
| *XC_2302* | *cheY* | chemotaxis response regulator | +3.41↑ | 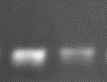 |
| *XC_2237* | *flgI* | flagellar protein | +2.91↑ | 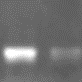 |
| *XC_2282* | *cheY* | chemotaxis protein | +3.86↑ | 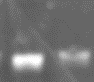 |
| *XC_2723* |  | transcriptional regulator | +2.45↑ | 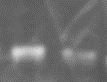 |
| *XC_1887* |  | ABC transporter ATP-binding protein | -2.08↓ | 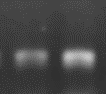 |
| *XC_0820* | *dctA* | C4-dicarboxylate transport protein | +2.6↑ | 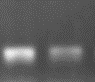 |
| *XC_0924* | *fhuE* | outer membrane receptor for ferric iron uptake | +2.23↑ | 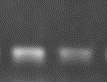 |
| *XC_1119* | *bla* | beta lactamase | +3.53↑ | 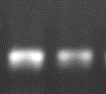 |
| *XC_0705* | *peh* | endopolygalacturonase | +2.13↑ | 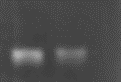 |
| *XC_3001* | *hpa2* | Hpa2 protein | +2.33↑ | 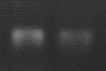 |
| *XC_2821* |  | conserved hypothetical protein | -2.36↓ | 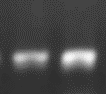 |
| *XC_3422* |  | conserved hypothetical protein | -2.19↓ | 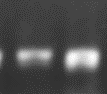 |
| *XC_0817* |  | conserved hypothetical protein | +2.31↑ | 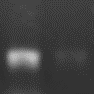 |
| *XC_2631* |  | conserved hypothetical protein | +2.35↑ | 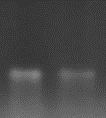 |
| *XC_3461* |  | conserved hypothetical protein | +3.39↑ | 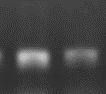 |
| 16S |  |  |  | 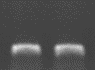 |

Table S5. Primers used in this study

| Primer | Nucleotide sequence (5′→3′) | The amplified fragment or the utilization |
| --- | --- | --- |
| L1304F  L1304R | CGCGGATCCTGCTGCGGGTGATGAACTAC  TGCTCTAGACAGACGCTGCAGCTGCGTTT | 555-bp DNA sequence upstream of *ptsI*, used for construction of *ptsI* deletion mutant. |
| R1304F  R1304R | TGCTCTAGACAAGGCCTGCTGCACTGAGT  CCCAAGCTTGTACAGGTGATCGGTGTGGT | 692-bp DNA sequence downstream of *ptsI*, used for construction of *ptsI* deletion mutant. |
| C1304F  C1304R | CGCGGATCCAAGAGCATCATGGGTGTGAT  CCCAAGCTTAGCAGGGCATTATCGCTTGC | 2015-bp DNA fragment spans 195 upstream to 89 bp downstream of the *ptsI* ORF sequence. |
| L1305F  L1305R | CGCGGATCCCACACTGACCAAAGCGCTAC  TGCTCTAGACATCGTCGATGATCGCTCCG | 679-bp DNA sequence upstream of *ptsH*, used for construction of *ptsH* deletion mutant. |
| R1305F  R1305R | TGCTCTAGAAGGCGTTGGTGGACCTGTTC  CCCAAGCTTTTGTCGCACACCAGGATCTC | 593-bp DNA sequence downstream of *ptsI*, used for construction of *ptsH* deletion mutant. |
| CptsH-F  CptsH-R | CGCGGATCCATGCTTGAACGCGAACTC  CCCAAGCTTTCAGCTGTCCTCGTCGAA | 270-bp DNA fragment of *ptsH* coding sequence. Used for complementary assay and overproduction of HPr protein. |
| ptsH-1F  ptsH-1R | GGGGTACCGCTTGAACGCGAACTCATAGT  TGCTCTAGATCAGCTGTCCTCGTCGAA XbaI | 270-bp DNA fragment of *ptsH* coding sequence. Used for overexpression of *ptsH* gene in *Xcc* strain. |
| ptsH-2F  ptsH-2R | CGCGGATCCATGCTTGAACGCGAACTCATA TGCTCTAGATCAATGATGATGATGATGGTGGCTGTCCTCGTCGAAGC | 288-bp DNA fragment of *ptsH* coding sequence fused with 6×His tag coding sequence. Used for western blotting assay. |
| L1306F  L1306R | CGCGGATCCAGGATTGAATCTCGGGATTG  TGCTCTAGACGGTCTTCAACGGCAAATGC | 616-bp DNA sequence upstream of *ptsN^Man^*, used for construction of *ptsN^Man^* deletion mutant. |
| R1306F  R1306R | TGCTCTAGAGCAACGGAGCGATCATCGAC  CCCAAGCTTACAGGGCATGCAGTTCGAGG | 593-bp DNA sequence downstream of *ptsN^Man^*, used for construction of *ptsN^Man^* deletion mutant. |
| C1306F  C1306R | CGCGGATCCTCACATCGTCACGCTGTCAG  CCCAAGCTTAGGACGACAGCGTCTGCACC | 612-bp DNA fragment spans 142 upstream to 77 bp downstream of the *ptsN^Man^* ORF sequence. |
| L1308F  L1308R | CCGGAATTCGTCCGCAAGCCAGACCAC  TGCTCTAGAAGTGCCAGCTTGTCGCGC | 726-bp DNA sequence upstream of *hprK_Xcc_*, used for construction of *hprK_Xcc_* deletion mutant. |
| R1308F  R1308R | TGCTCTAGACCGGCGGCGATGTTCATC  CCCAAGCTTAGCTGCGGGTGTCGTTGC | 798-bp DNA sequence downstream of *hprK_Xcc_*, used for construction of *hprK_Xcc_* deletion mutant. |
| ChprK-F  ChprK-R | CGCGGATCCTGGACCTGGTGTTTGCGA  CCCAAGCTTGTCTTCAGCGCCACCGAC | 1201-bp DNA fragment spans 180 upstream to 70 bp downstream of the *hprK_Xcc_* ORF sequence. |
| OhprK-F  OhprK-R | GGGGATCCATGAATACCAGCATCACCGCA  GGAAGCTTTCATGGGGTGCGTCGCTCCAG | 948-bp DNA fragment of the *hprK_Xcc_* coding sequence. Used for overproduction of HprK_Xcc_. |
| L1309F  L1309R | CGCGGATCCCCAAGTTGGCAGATCTGTTG  TGCTCTAGAGCATGAGGGCATCAAGCGAG | 545-bp DNA sequence upstream of *ptsN^Ntr^*, used for construction of *ptsN^Ntr^* deletion mutant. |
| R1309F  R1309R | TGCTCTAGAGCTGATGGCGCTGCTGACTG  CCCAAGCTT CAGGTGCCGTCGAGTACGTC | 641-bp DNA sequence downstream of *ptsN^Ntr^*, used for construction of *ptsN^Ntr^* deletion mutant. |
| C1309F  C1309R | CGCGGATCCACTTCGACCAGCACTGTGAA  CCCAAGCTTCGTGCGGTGATGCTGGTATT | 728-bp DNA fragment spans 250 upstream to 19 bp downstream of the *ptsN^Ntr^* ORF sequence. |
| HptsH-F  HptsH-R | AATCGACTGGGACTGGCTGCGCGGGCAACCG  CGGTTGCCCGCGCAGCCAGTCCCAGTCGATT | Use for creating HPr protein point mutant with alanine replaces histidine at position 15. |
| SptsH-F  SptsH-R | CGAAGTGAATGCCAAGGCTATCATGGGTGTGATGC  GCATCACACCCATGATAGCCTTGGCATTCACTTCG | Use for creating HPr protein point mutant with alanine replaces serine at position 46. |
| 1305N-F  1305N-R | TgCTTgAACgCgAACTCATAgTT  TCACACCCATgATgCTCTTgg | 150-bp ptsH internal fragment, used for RT-PCR to examine the expression level of HPr protein. |
| 1575-F  1575-R | CAGGAACTGTCTGCCGCCTA  CTCGGCAATGATGCGGGTCT | 255-bp DNA fragment spans nucleotides 106 to 360 bp of the *XC_1575*, used for RT-PCR. |
| 2375-F  2375-R | GTCGCATCATTCCCTGCCTG  ATGTCGATCAGGCGCGCAAC | 214-bp DNA fragment spans nucleotides 11 to 224 bp of the *XC_2375*, used for RT-PCR. |
| 0970-F  0970-R | CCAATCCAAGGGTGACTGGT  TGTTGATGTCGTGCTCGAAT | 200-bp DNA fragment spans nucleotides 69 to 268 bp of the *XC_0970*, used for RT-PCR. |
| 1621-F  1621-R | CGGGTTCACGCTGGTTGAGT  GCCGTTACTGCTTCCACAGA | 202-bp DNA fragment spans nucleotides 21 to 222 bp of the *XC_1621*, used for RT-PCR. |
| 2302-F  2302-R | ATCTTGGTGGTGGACGATTC  ACTTGTAGTCCGGCAGCTGG | 214-bp DNA fragment spans nucleotides 13 to 126 bp of the *XC_2302*, used for RT-PCR. |
| 0993-F  0993-R | GAGGCCGAGAGCATCCACAT  TTGATGTGCACGCGCAGGTC | 239-bp DNA fragment spans nucleotides 37 to 275 bp of the *XC_0993*, used for RT-PCR. |
| 3740-F  3740-R | CCTTTTATGGCGATCGCAGC  ACGGATGGGTCGTTGGGATC | 208-bp DNA fragment spans nucleotides 74 to 281 bp of the *XC_3740*, used for RT-PCR. |
| 2723-F  2723-F | TGGATCTGGAAGACTGGTCG  CGTCAAACCGGTAGTAGGCC | 219-bp DNA fragment spans nucleotides 59 to 277 bp of the *XC_2723*, used for RT-PCR. |
| 1887-F  1887-R | GATGTGTTTACCCGCCACCT  TCAGTTTCTCGCCGTAGCGC | 226-bp DNA fragment spans nucleotides 31 to 256 bp of the *XC_1887*, used for RT-PCR. |
| 3560-F  3560-R | CGCCGTTGTGATCAACCTGC  TCACGTTGCGGCATTGGCAG | 237-bp DNA fragment spans nucleotides 21 to 257 bp of the *XC_3560*, used for RT-PCR. |
| 2709-F  2709-R | CGTGCCGATCGCCTGTTCAA  TCAGCGCAGTGACCAAGGTG | 211-bp DNA fragment spans nucleotides 64 to 274 bp of the *XC_2709* (*pstC*), used for RT-PCR and Real-time quantitative PCR. |
| 1350-F  1350-R | TCGCGACCAGTGGCTTCGAA  TTGATCACACCGCCTGGCAG | 217-bp DNA fragment spans nucleotides 38 to 254 bp of the *XC_1350*, used for RT-PCR. |
| 0556-F  0556-R | CTGGCCCAGGACACCTTCAT  TCCTTCACTTTCGGCGGCAG | 260-bp DNA fragment spans nucleotides 118 to 277 bp of the *XC_0556*, used for RT-PCR. |
| 0557-F  0557-R | GTGGCTGACGGTGCTGATGT  AGCACCAGGTTGAGCGCCTT | 228-bp DNA fragment spans nucleotides 21 to 248 bp of the *XC_0557*, used for RT-PCR. |
| 0028-F  0028-R | CGCGGCGGATTGAAGTACGT  GCATCCAGCTCGCCATTGAG | 200-bp DNA fragment spans nucleotides 106 to 305 bp of the *XC_0028* (*egl*), used for RT-PCR and Real-time quantitative PCR. |
| 0738-F  0738-R | CACTGCCGCTGTCTGTGTGT  ATCGCCGGTACTCAGGTACG | 244-bp DNA fragment spans nucleotides 54 to 297 bp of the *XC_0738* (*xcsC*), used for RT-PCR and Real-time quantitative PCR. |
| 1658-F  1658-R | CTGGCAATGTCCACGGTGCA  TAGCCAGCGCGATACCGATC | 209-bp DNA fragment spans nucleotides 64 to 272 bp of the *XC_1658* (*gumB*), used for RT-PCR and Real-time quantitative PCR. |
| 2699-F  2699-R | GACACAACGTCCGAGAAGGG  TCACGCTTCTGGAACGCTTC | 200-bp DNA fragment spans nucleotides 67 to 266 bp of the *XC_2699*, used for RT-PCR. |
| 3376-F  3376-R | GTGTGTGTGTGCGATGGCGT  TCAGGCTTTCCGAGTCGACC | 284-bp DNA fragment spans nucleotides 123 to 406 bp of the *XC_3376* used for RT-PCR. |
| 3754-F  3754-R | GCCATGCGCTACTTCACCCA  TGGTGCTGTTCTGGGTCATG | 284-bp DNA fragment spans nucleotides 115 to 316 bp of the *XC_3754* used for RT-PCR. |
| 2857-F  2857-R | CATCGCGACAGCAGCAGACA  TGGTCACATCGTTGGCCGTC | 230-bp DNA fragment spans nucleotides 120 to 349 bp of the *XC_2857*used for RT-PCR. |
| 2237-F  2237-R | CGCCGAGTTTGCCGACATGT  GCATCGGTCTGGAAGTTGCC | 222-bp DNA fragment spans nucleotides 108 to 329 bp of the *XC_2237* used for RT-PCR. |
| 2282-F  2282-R | CGGATCCTGATCGTGGACGA  AGGTGCTTGAGCTTGGCGTC | 224-bp DNA fragment spans nucleotides 4 to 227 bp of the *XC_2282* used for RT-PCR. |
| 0820-F  0820-R | GCCTTCGCCGAAAGCCTCAA  ACCACCATGCCGACGATCAA | 200-bp DNA fragment spans nucleotides 118 to 317 bp of the *XC_0820* used for RT-PCR. |
| 0924-F  0924-R | CTGAGCGTGGGCTACGAGTA  CTTGAGCGTCCAGCCGTTGT | 201-bp DNA fragment spans nucleotides 118 to 318 bp of the *XC_0924* used for RT-PCR. |
| 1119-F  1119-R | CGGTGGTGAACGTGGACGAT  CGGATCTTGATCTGCTGCGT | 229-bp DNA fragment spans nucleotides 83 to 311 bp of the *XC_11196* used for RT-PCR. |
| 0705-F  0705-R | AGTTCGCGGCGGCATTGGAA  GGCATCCGCCACTCTTGGAA | 252-bp DNA fragment spans nucleotides 161 to 412 bp of the *XC_0705* used for RT-PCR. |
| 3001-F  3001-R | CTCGCACGCGCAGACTGTTT  CGCATCCCTGGAGATACCGT | 204-bp DNA fragment spans nucleotides 34 to 273 bp of the *XC_3001* used for RT-PCR. |
| 2821-F  2821-R | GGTGCCGTTTGCCGAGAAAA  CACCTGCGCCATGCAATTGG | 226-bp DNA fragment spans nucleotides 45 to 270 bp of the *XC_2821* used for RT-PCR. |
| 3422-F  3422-R | CACACCGAAGAACGCCTGCT  ACTGAAACCGATGGTGGCGC | 222-bp DNA fragment spans nucleotides 118 to 339 bp of the *XC_3422* used for RT-PCR. |
| 0817-F  0817-R | CAAGGTGGACGCCCGATGAT  ATGTGGCCGGTGCTGAGATG | 218-bp DNA fragment spans nucleotides 16 to 233 bp of the *XC_0817* used for RT-PCR. |
| 2631-F  2631-R | AACCTAAGACACCGGCCACG  CAGCTTCAACGCCTTCGCTA | 200-bp DNA fragment spans nucleotides 8 to 207 bp of the *XC_2631* used for RT-PCR. |
| 3461-F  3461-R | GCGAGTACGCCGTGGACTTT  TCATAGGTCCAGTCCTGCCG | 202-bp DNA fragment spans nucleotides 119 to 320 bp of the *XC_3461* used for RT-PCR. |
| 1298-F  1298-R | CGGCAAGTTCGACTTCGGCA  ATCTTCGCCGCCCTGCAACA | 205-bp DNA fragment spans nucleotides 201 to 405 bp of the *XC_1298* (*pelB*) used for Real-time quantitative PCR. |
| 0851-F  0851-R | ACGTCGTCATCGAGATCCCC  TCATCGCTCATGCGCAGCAC | 247-bp DNA fragment spans nucleotides 50 to 296 bp of the *XC_0851* (*virP*) used for Real-time quantitative PCR. |
| 0153-F  0153-R | GGCTATCAGCGCATCGTGCT  GCATCGATGCCATAGCGCGT | 200-bp DNA fragment spans nucleotides 283 to 482 bp of the *XC_0153* (*xynB*) used for Real-time quantitative PCR. |
| 3657-F  3657-R | TAGCTCCACTTCGGTATCCG  TGGTGTGCCGAGTCCACTGT | 222-bp DNA fragment spans nucleotides 219 to 440 bp of the *XC_3657* (*copB*) used for Real-time quantitative PCR. |
| 4064-F  4064-R | GTTGTTCGCCTACGCGTTCG  ATTGACCAGCAGGCCGACGA | 217-bp DNA fragment spans nucleotides 210 to 426 bp of the *XC_4064* (*czcD)* used for Real-time quantitative PCR. |
| 0939-F  0939-R | GCTCAGCGATCTGGACTTCA  TAAGCGGCTCCAGATTCACC | 215-bp DNA fragment spans nucleotides 21 to 235 bp of the *XC_0939* (*pilO*) used for Real-time quantitative PCR. |
| 16S-F  16S-R | GCCTAACACATGCAAGTCGAACGGC  AATATTCCCCACTGCTGCCTCCCG | 325-bp DNA fragment of the 16S rDNA sequence, used for RT-PCR and Real-time quantitative PCR |

§The underlined sequences indicate the restriction sites for *Bam*HI, *Hin*dIII, *Kpn*I and *Xba*I, respectively.
